# BHLHE40 Orchestrates Effector Tissue‐Resident Memory CD8^+^ T Cells and Limits Long‐Term Survival of Kidney Graft

**DOI:** 10.1002/advs.202520518

**Published:** 2026-01-04

**Authors:** Junbo Li, Bo Yang, Tianhui Pan, Xi Zhou, Zhibo Ma, Du Tang, Bowen Xie, Jing Liu, Zhishui Chen, Peixiang Lan

**Affiliations:** ^1^ Institute of Organ Transplantation Tongji Hospital Tongji Medical College Huazhong University of Science and Technology Key Laboratory of Organ Transplantation Ministry of Education NHC Key Laboratory of Organ Transplantation Chinese Academy of Medical Sciences Wuhan Hubei P. R. China; ^2^ State Key Laboratory for Diagnosis and Treatment of Severe Zoonotic Infectious Diseases Huazhong University of Science and Technology Wuhan Hubei P. R. China; ^3^ Division of Cardiology Department of Internal Medicine Tongji Hospital Tongji Medical College Huazhong University of Science and Technology Wuhan Hubei P. R. China

**Keywords:** BHLHE40, Tissue‐resident memory CD8^+^ T cells, kidney graft, tertiary lymphoid structures

## Abstract

Tissue‐resident memory T cells (T_RM_), which function against tumors, infections, and non‐self antigens in organ transplantation, exhibit both effector and memory functionality. However, the homeostasis and differentiation of T_RM_ is not clear. Using a murine kidney transplant model for long‐term observation, our single‐cell and spatial transcriptomics showed that CD49a^+^PD1^hi^ CD8^+^ T_RM_ exhibited an effector phenotype with enhanced cytotoxicity at a later stage. This subset might mature from a CXCR6^hi^ precursor‐like state with proliferation capacity in tertiary lymphoid structures (TLSs) in allografts. Mechanically, BHLHE40 is required for CD49a^+^PD1^hi^ CD8^+^ T_RM_ differentiation and effector function, thereby driving rejection in allo‐transplantation. In TLSs, TGF‐β orchestrated BHLHE40 expression in T_RM_ and effector T_RM_ differentiation. Our findings identified an effector subset of T_RM_ as CD49a^+^PD1^hi^ CD8^+^ T_RM_, and highlighted a BHLHE40‐orchestrated, resident immune component, rather than circulating cells, as a major contributor to allograft rejection.

## Graphical abstract

An effector subset of T_RM_, defined as CD49a^+^PD1^hi^ CD8^+^ T_RM_, represents a BHLHE40‐orchestrated resident immune component that, rather than circulating cells, plays as a major contributor to allograft rejection.

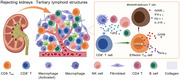

## 1 Introduction

In tumor tissues and infection, only part of T cells recognize specific antigens presented by self‐major histocompatibility complex (MHC) molecules by T cell receptor (TCR) [1, 2]. Unlike T cell response to conventional antigens, most of T cells directly recognize nonself MHC molecules in allogeneic cell and organ transplantation, supported by the observation that T cell response to allogeneic MHC molecules is quick and strong in the mixed lymphocyte reaction in 1963 [3, 4, 5]. One proposed reason is that TCR from one individual recognizes conserved regions of the allogeneic MHC protein as nonself [5]. MHC incompatibility provokes rapid and strong T cell activation, leading to rapid graft rejection in transplant [6]. However, it is unknown how T cells differentiate and form memory T cells when the MHC is incompatible in transplantation. Although lots of studies focus on T cell responses to specific antigens presented by self‐MHC molecules.

In the TCR‐self MHC setting, such as chronic infections, cancer, and autoimmune diseases, antigen‐specific naive T cells recognize antigens and undergo activation, proliferation, and differentiation into effector T cells [7]. Some antigen‐specific long‐lived T cells can response strongly to specific antigen stimulation, called memory T cells [8, 9]. Emmerging evidence suggests that tissue‐resident memory T cells (T_RM_) cells play a pivotal role in long term protection against infection and tumor progress [10]. T_RM_ cells, regulated by cellular interactions and chemokines such as CD103 (*Itgae*) and CXCR6, exhibit transcriptional heterogeneity, with a subset characterized by high expression of effector molecules like granzyme B and interferon‐γ [10, 11, 12, 13]. However, the heterogeneity of T_RM_ cells, their specific markers in the context of kidney transplantation in the TCR‐non‐self MHC setting, and the transcriptional programs driving their pathogenic effector functions remain areas of active investigation.

Kidney transplantation is the most effective treatment for end‐stage renal disease, yet long‐term allograft survival remains a significant challenge, with chronic rejection being a primary cause of late allograft loss [14, 15]. While acute rejection, often mediated by circulating effector T cells which are activated by nonself MHC antigens, can be effectively managed with current immunosuppressive therapies, chronic rejection is an insidious process involving complex immunological and non‐immunological mechanisms that lead to progressive graft dysfunction and eventual failure [16, 17, 18, 19]. Increasing evidence suggests that, under the persistent stimulation of alloantigens and chronic inflammation, T_RM_ cells can arise within the allograft microenvironment and play a pivotal role in the initiation and progression of chronic rejection [20, 21]. These cells exhibit unique transcriptional profiles and tissue‐specific functional properties, contributing not only to protective immunity but also to immune‐mediated tissue damage [11, 22, 23]. Furthermore, unlike tumor‐bearing mouse models where the observation period is typically restricted to less than one month [24, 25, 26], the murine kidney transplantation model allows for long‐term longitudinal observation (exceeding 60 days). This extended temporal window enables a more comprehensive assessment of the evolving immune landscape, particularly allowing for the investigation of the long‐term homeostasis, plasticity, and functional maturation of T_RM_ cells during the progression of chronic rejection.

The allograft microenvironment is a dynamic landscape capable of generating organized immune aggregates, known as tertiary lymphoid structures (TLSs), in response to chronic inflammation and persistent antigen stimulation‐ectopic lymphoid formations that arise in chronic infections and autoimmune diseases. Notably, in chronically rejected solid organ transplants, they serve as local hubs for immune cell priming, differentiation, and antibody production [27, 28, 29]. Despite their association with poor graft outcomes and heightened alloimmunity in rejected kidney allografts [30, 31], the extent to which TLSs provide essential niches and instructive cues for the development and persistence of CD8^+^ T_RM_ cells remains unclear and warrants further investigation.

By integrating single‐cell and spatial transcriptomics with functional studies in a murine kidney transplant model and human samples, we aim to delineate the characteristics of allograft T_RM_ cells, identify their key regulatory mechanisms, and explore their contribution to chronic rejection. Our findings reveal BHLHE40 as a central orchestrator of CD8^+^ T_RM_ cell differentiation and effector function, partly driven by local TGF‐β signaling within graft‐associated TLSs, ultimately promoting chronic allograft injury. This research not only elucidates a novel mechanism underlying transplant rejection but also identifies BHLHE40 and associated pathways as potential therapeutic targets to improve long‐term graft survival.

## 2 Results

### 2.1 Persistent Antigen Exposure Drives Memory Phenotype Acquisition in Graft‐Infiltrating CD8^+^ T Cells

**FIGURE 1:**
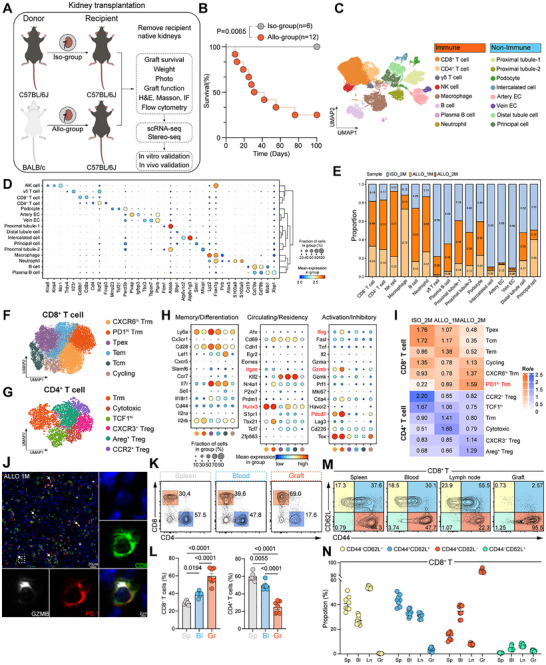
Persistent antigen exposure induces memory‐like differentiation in graft CD8^+^ T cells. (A) Study design for murine kidney transplantation model characterization. Schematic outlining the establishment of syngeneic (Iso‐group; C57BL/6J→C57BL/6J) and allogeneic (Allo‐group; BALB/c→C57BL/6J) kidney transplant models. Subsequent analyses listed represent the workflow for model characterization, including transcriptomics (scRNA‐seq, Stereo‐seq) and downstream validation studies. (B) Survival analysis of Allo‐group (*n* = 12) versus Iso‐group (*n* = 6) kidney transplant recipients. (C) UMAP plot of 45,252 cells profiled by scRNA‐seq colored by cell type in Iso (day 60) and Allo (day 30 and day 60) groups. (D) Dot plot showing average expression (color) and percentage of expressing cells (dot size) for selected genes across clusters. (E) Cell type composition across samples. (F,G) UMAP plots of CD8^+^ (*n* = 16,142 cells) (F) and CD4^+^ (*n* = 3,840 cells) (G) T cells colored by subcluster. (H) Dot plot of selected genes associated with memory/differentiation (left), circulation/residency (center), and activation/inhibition (right) in CD8^+^ T cell subclusters (F). (I) Relative enrichment of each cluster across groups, measured by the ratio of observed to expected cell numbers (Ro/e). (J) Representative immunofluorescence images of CD8, PD‐1 and GZMB staining in graft sections. Scale bars, 20 and 2 µm. (K,L) Flow cytometry analysis of CD8^+^ and CD4^+^ T cell frequencies in spleen (sp), blood (bl), and graft (gr) from Allo‐group mice (Day 30). (M,N) CD44 and CD62L expression defining Naive (CD44^−^CD62L^+^), Tcm (CD44^+^CD62L^+^), and Tem (CD44^+^CD62L^−^) subsets within CD8^+^ T cells across tissues from Allo‐group mice. Data are mean ± s.e.m. of biologically independent samples. *p*‐values are from a two‐tailed unpaired Student's *t*‐test (L) and log‐rank test (B).

To investigate the dynamic responses of T cells in the setting of MHC incompatibility during the progression of chronic rejection (up to 100 days), we established a fully MHC‐mismatched (BALB/c to C57BL/6, Allo‐group) and syngeneic (C57BL/6 to C57BL/6, Iso‐group) mouse kidney transplantation model (Figure 1A). Recipient mice underwent bilateral nephrectomy at the time of transplantation, rendering their survival dependent on the transplanted kidney. As depicted in the survival curves, in the absence of any intervention, mice in the Allo‐group progressively succumbed to rejection starting from day 7, whereas the Iso‐group exhibited 100% survival at day 100 (Figure 1B). Compared to the Iso‐group, the Allo‐group exhibited a significant increase in serum creatinine levels at 30 d post‐transplantation, suggesting potential renal impairment (Figure S1A,B). In addition, a marked postoperative weight loss was observed in the Allo‐group mice, which may reflect a heightened systemic inflammatory response associated with allogeneic immune activation (Figure S1C). To further evaluate graft pathology, we examined the transplanted kidneys at 30 d post‐transplantation. Kidneys from the Allo‐group appeared grossly more swollen compared to those from the Iso‐group, with significantly increased graft weight (Figure S1D,E).

To examine the evolving landscape of immune cells within the kidney allograft, we performed single‐cell transcriptomic analyses on graft tissues collected from Allo‐group recipients at 1 month (ALLO_1 m) and 2 months (ALLO_2 m) post‐transplantation, as well as from Iso‐group recipients at 2 months (ISO_2 m). We integrated 45 252 high‐quality cells from three samples using a deep learning framework (scVI) to correct for technical variations and performed unbiased clustering (Figure S2A) [32]. Uniform Manifold Approximation and Projection (UMAP) and clustering analyses identified 8 immune cell clusters and 8 non‐immune cell clusters (Figure 1C). Cell‐type annotation was aided by unsupervised mapping to a reference atlas and further validated by manual inspection of marker gene expression, as well as by transcriptomic similarity analysis using correlation matrices (Figure 1D; Figure S2B–D). Quantification of cellular composition in ALLO_1 m and ALLO_2 m grafts relative to ISO_2 m revealed a higher abundance of immune cells in the allogeneic grafts, particularly T cells (Figure 1E). We conducted a more in‐depth clustering analysis on the identified CD8^+^ and CD4^+^ T cell clusters with the aim of discovering and characterizing subpopulations with distinct gene expression profiles. We observed 12 distinct T cell states, including 6 transcriptionally defined CD8^+^ subsets: CXCR6^hi^ T_RM_, PD1^hi^ T_RM_, progenitor exhausted T cells (Tpex), effector memory T cells (Tem), central memory T cells (Tcm), and Cycling, and 6 CD4^+^ subsets comprising T_RM_, Cytotoxic, TCF1^hi^, and Tregs characterized by CXCR3^+^, Areg^+^, and CCR2^+^ Tregs (Figure 1F,G; Figure S2E). Notably, CD8 T_RM_ cells (including CXCR6^hi^ T_RM_ and PD1^hi^ T_RM_) exhibited high expression of inflammatory cytokines *Ifng* and *Gzmb*, as well as exhaustion markers *Pdcd1* and *Tox*, while showing minimal expression of *Tcf7*, *S1pr1*, *Il7*, and *Klf2* (Figure 1H). Relative enrichment analysis based on the ratio of observed to expected cell numbers revealed a significant enrichment of PD1^hi^ T_RM_ cells in the ALLO group (Figure 1I). Representative immunofluorescence images of Allo‐group kidney grafts at Day 30 post‐transplantation, showing staining for CD8 (green), PD‐1 (red), and GZMB (white). (Figure 1J). Flow cytometric analysis was performed to characterize T cell populations in the spleen, peripheral blood, and kidney grafts of Allo‐group mice at 30 d post‐transplantation. Flow cytometry revealed a progressive increase in CD8^+^ T cell proportions from the spleen to peripheral blood and ultimately to the graft, where they markedly outnumbered CD4^+^ T cells, suggesting a dominant role of CD8^+^ T cells in the local immune response (Figure 1K,L; Figure S2F). Compared to peripheral blood, mesenteric lymph nodes, and spleen, graft‐infiltrating CD4^+^ and CD8^+^ T cells were overwhelmingly enriched for CD44^+^CD62L^−^ effector memory cells, with a near‐complete absence of CD44^−^CD62L⁺ naïve T cells (Figure 1M,N; Figure S2G,H). Collectively, this suggests that T cells that have undergone the acute rejection phase were predominantly converted into memory T cell populations, highlighted by the prominent formation of T_RM_, which may continue to mediate ongoing allogeneic immune responses within the graft.

### 2.2 Tertiary Lymphoid Structures Localize with Immune Activation Zones in Kidney Allografts

**FIGURE 2:**
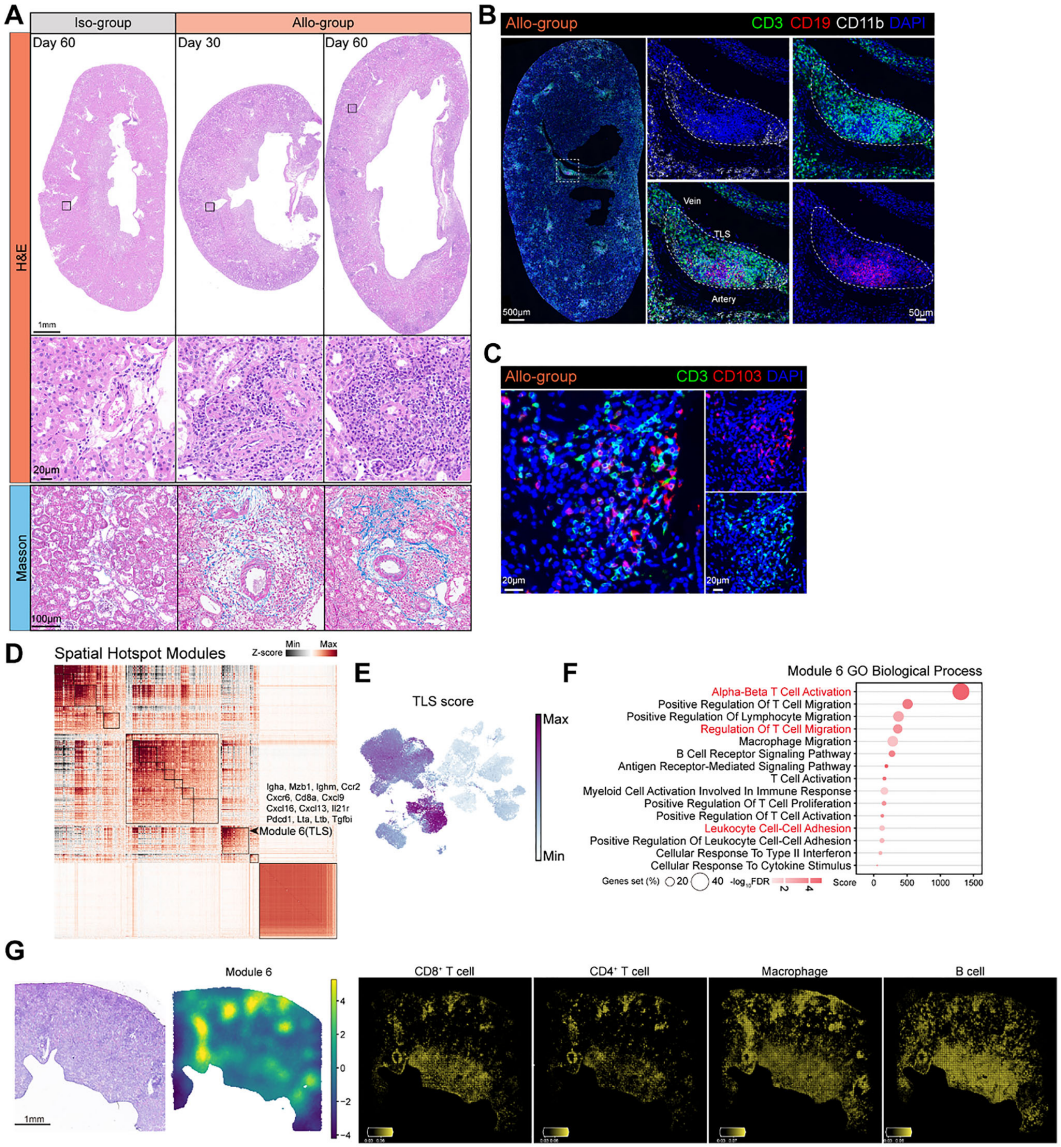
Tertiary lymphoid structures localize with immune activation zones in kidney allografts. (A) Representative H&E and Masson's trichrome staining of Iso‐ (Day 60) and Allo‐group (Day 30, Day 60) grafts. Scale bars, 1 mm, 20 and 100 µm. (B) Immunofluorescence of an Allo‐group graft (Day 30) showing a TLS (dashed line) with CD3^+^ (green), CD19^+^ (red), and CD11b^+^ (white) cells. Scale bars, 500 and 50 µm. (C) Immunofluorescence showing CD3^+^ (green) T cells co‐expressing CD103 (red, arrows) in an Allo‐group graft (Day 30). Scale bars, 20 µm. (D), Heatmap of genes exhibiting significant spatial autocorrelation, clustered into distinct modules according to spatial hotspots detected in graft tissue sections (section ALLO_1 m). (E,F) UMAP visualization of Module 6 activity scores (E), and GO enrichment analysis of Module 6 genes (F). (G) H&E (left) and spatial expression of Module 6 genes on graft tissue sections (middle), and spatial distribution of CD8^+^ T cell, CD4^+^ T cell, macrophages, and B cells across grafts inferred by Cell2location (right). Scale bars, 1 mm.

Histological analyses using hematoxylin and eosin (H&E) and Masson's trichrome staining revealed substantial mononuclear cell and lymphocyte infiltration in the renal interstitium of the Allo‐group (Figure 2A). In addition, vascular changes such as intimal hyperplasia were observed, along with prominent deposition of blue‐stained collagen fibers, indicating extensive fibrosis (Figure 2A). Histopathological analysis revealed prominent immune cell aggregates surrounding the perivascular regions of the allografts. Immunofluorescence staining identified these clusters as being composed of CD3^+^ T cells, CD19^+^ B cells, and CD11b^+^ myeloid‐derived cells (Figure 2B), characteristic of tertiary lymphoid structures (TLSs). Notably, these immune aggregates exhibited high expression of Ki‐67, indicating active cellular proliferation within these regions (Figure S3A). Immunofluorescence staining for CD3 and CD103 revealed that a subset of CD3^+^ T cells within the graft expressed CD103, suggesting the presence of T_RM_ populations in the allograft (Figure 2C).

To investigate the spatial organization of the immune microenvironment with subcellular resolution, we performed Spatial Enhanced Resolution Omics sequencing (Stereo‐seq) on kidney allograft sections from the ALLO_1 m and ALLO_2 m groups (matched to the scRNA‐seq timepoints). Utilizing this original high‐definition spatial transcriptomic dataset, we applied Hotspot to identify spatially co‐varying gene modules based on Stereo‐seq transcriptomic data [33]. Module 6, enriched in TLS‐associated genes including *Lta*, *Ltb*, *Pdcd1*, *Il21r*, *Cxcl9*, *Cxcl13*, *Igha*, and *Tgfbi*, was spatially and transcriptionally distinct from other modules (Figure 2D; Figure S3B). Gene scoring using Module 6 in scRNA data, followed by pathway enrichment analysis, showed that these TLS‐associated genes were predominantly expressed by immune cells and were enriched in immune activation‐related pathways (Figure 2E,F). Spatial mapping of single‐cell data using cell2location identified a higher abundance of CD4^+^ T cells, CD8^+^ T cells, B cells, and macrophages in regions corresponding to TLSs (Figure 2F; Figure S3C,D) [34].

### 2.3 CD49a Expression Delineates CD8^+^ and CD4^+^ T_RM_ Cells in Allorejection

**FIGURE 3:**
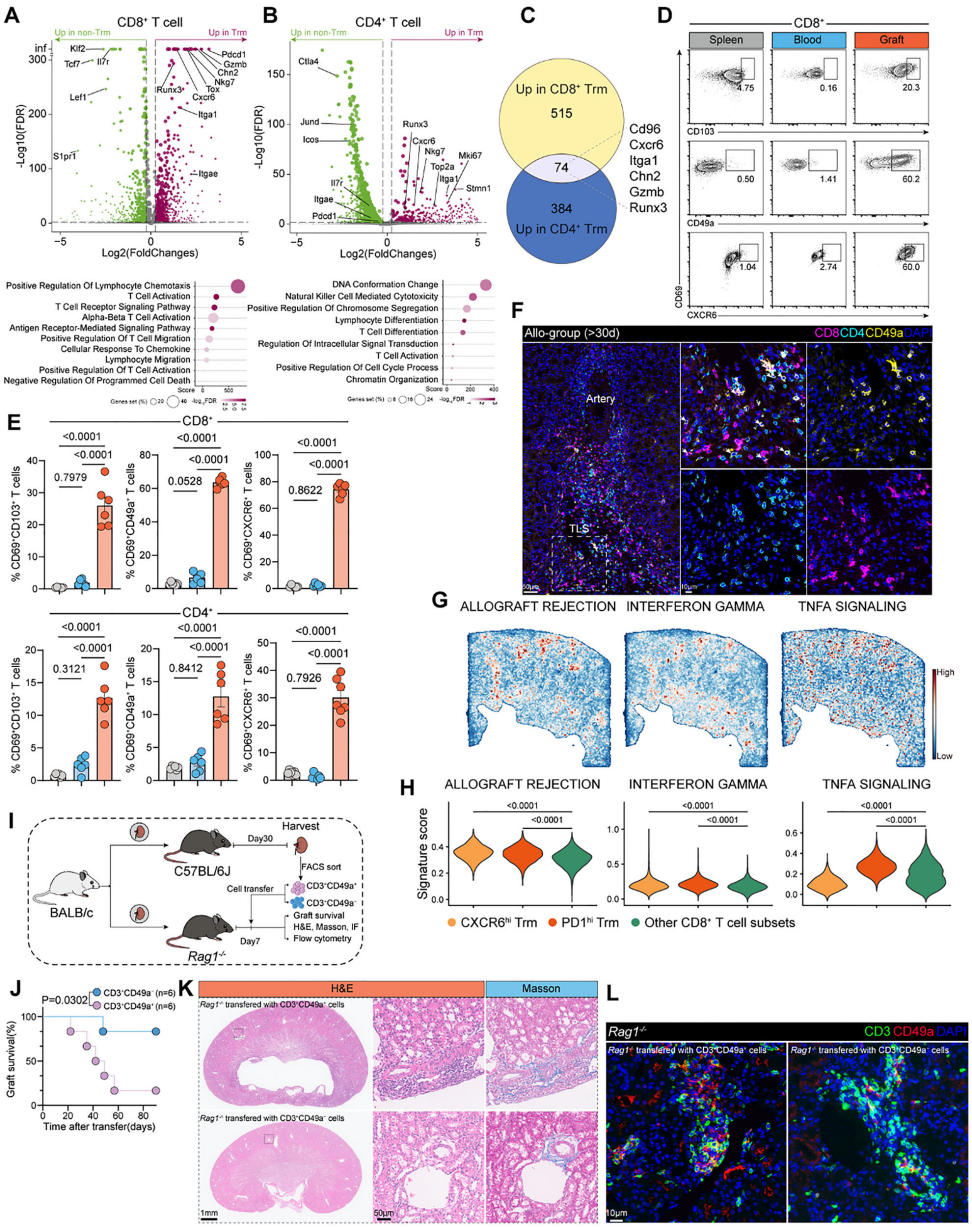
CD49a expression delineates CD8^+^ and CD4^+^ T_RM_ cells in allorejection. (A,B) Volcano plot comparing CD8^+^ or CD4^+^ T_RM_ (dark red upregulated) vs non‐T_RM_ (green upregulated) cells (top). Bottom: GO Biological Process enrichment for T_RM_ ‐upregulated genes (dot size: gene %; color: ‐log_10_ FDR). (C) Venn diagram illustrating the overlap between genes upregulated in CD8^+^ T_RM_ cells (vs non‐T_RM_) and genes upregulated in CD4^+^ T_RM_ cells (vs non‐T_RM_). (D,E) Flow cytometric analysis (D) and the corresponding statistical analysis (E) of CD8^+^ T_RM_ cells derived from spleen, blood, and graft tissues of Allo‐group (*n* = 6) mice at 30 days post‐transplantation. (F) Representative immunofluorescence images of CD8, CD4, and CD49a staining in graft sections. Scale bars, 50 and 10 µm. (G,H) Selected MSigDB pathways were spatially mapped on the graft tissue sections (section ALLO_1 m) using PROGENy inference (G); violin plots showing gene signature scores for allograft rejection, interferon gamma, and TNFα signaling pathways across CD8^+^ T cell subsets (H). (I–L) 5 × 10^5^ CD49a^+^ or CD49a^−^ T cells were sorted from kidney allografts (Day 30 in C57BL/6J) and transferred into secondary *Rag1* ^−/−^ recipients bearing BALB/c allografts (Day 7). Experimental design for adoptive transfer (I). Percentage kidney allograft survival. *n* = 6 mice per group (J). Grafts were harvested 20 days after transplantation for analysis in (K) and (L). Representative H&E and Masson's trichrome staining (K). Scale bars, 1 mm and 50 µm. Immunofluorescence of CD3 and CD49a in grafts (L). Scale bars, 10 µm. Data are mean ± s.e.m. of biologically independent samples. *p*‐values are from a two‐tailed unpaired Student's *t*‐test (E), log‐rank test (J), and two‐tailed Wilcoxon rank‐sum test (H).

T_RM_ cells phenotypes, and gene‐expression signatures exhibit substantial heterogeneity, shaped by a combination of local tissue cues, persistent antigen exposure, and the surrounding inflammatory milieu. Using the scRNA‐seq dataset, we observed significant differences in gene expression profiles between T_RM_ and non‐T_RM_ subsets within both CD8^+^ and CD4^+^ T cells (Figure 3A,B; Figure S4A). CD8^+^ T_RM_ cells were enriched for pathways related to T cell activation, migration, adhesion, and resistance to programmed cell death, whereas CD4^+^ T_RM_ cells predominantly showed enrichment in activation and proliferation‐related pathways (Figure 3A,B). Analysis of upregulated genes revealed 74 overlapping transcripts between CD8^+^ and CD4^+^ T_RM_ populations, as illustrated by a Venn diagram. Representative shared genes included *Itga1*, *Cxcr6*, *Chn2*, *Runx3*, and *Gzmb* (Figure 3C). The dot plot compares the expression of integrin family members, chemokine receptors, and interleukin receptors in CD8^+^ T cells (Figure S4B). We next used flow cytometry to assess the expression of CXCR6, CD49a (*Itga1*), and CD103 (*Itgae*). Flow cytometry analysis revealed that the CD69^+^CD49a^+^ phenotype more accurately reflected the proportion of bona fide tissue‐resident cells (Figure 3D,E; Figure S4C). Notably, CD103 was detectable in the peripheral blood and spleen of naïve mice, whereas CD49a and CXCR6 were expressed at very low levels (Figure S4D). In contrast, reliance on CD69^+^CXCR6^+^ or CD69^+^CD103^+^ phenotypes alone tended to substantially overestimate or underestimate the actual frequencies of resident CD8^+^ or CD4^+^ T cells. Immunofluorescence staining revealed a substantial accumulation of T_RM_ cells within TLSs (Figure 3F).

We applied PROGENy to perform pathway scoring analysis on the spatial transcriptomics data [35]. The results revealed that the spatial distribution of the allograft rejection, interferon‐gamma, and TNFα signaling pathways was significantly altered, with these pathways preferentially enriched within TLSs (Figure 3G; Figure S4E). Pathway scoring based on these signatures showed that CD8^+^ T_RM_ cells, particularly PD1^hi^ T_RM_, exhibited significantly higher scores compared to non‐T_RM_ cells (Figure 3H).

### 2.4 CD49a^+^ T_RM_ Induces Graft Rejection

To evaluate the impact of T_RM_ cells on allograft survival, at 30 d post‐kidney transplantation, we isolated CD49a^+^ T cells and CD49a^−^ T cells from ALLO‐group recipients and separately adoptively transferred them into secondary *Rag1^−/−^* recipients bearing 7‐day‐old BALB/c kidney allografts (Figure 3I; Figure S4F). Compared to CD49a^−^ T cells, CD49a^+^ T cells preferentially localized to the transplanted kidney (Figure S4G,H). Adoptive transfer of CD49a^+^ T cells, but not CD49a^−^ T cells, was sufficient to induce kidney allograft rejection in *Rag1^−/−^* mice (Figure 3J). Adoptive transfer of CD49a^+^ T cells led to pronounced immune cell infiltration, exacerbated tissue injury, and marked fibrotic deposition within the kidney allografts (Figure 3K,L).

### 2.5 Transition of CD8^+^ T_RM_ Cells to Effector Phenotypes in Allografts

To further evaluate the specific T_RM_ subset responsible for mediating graft rejection, we isolated CD49a^+^ or CD49a^−^ CD8^+^ T cells via flow cytometry and separately adoptively transferred them into *Rag1^−/−^* mice (Figure S5A,B). *Rag1^−/−^* recipients receiving CD49a^+^CD8^+^ T cells exhibited progressive graft rejection and eventual death by day 40 post‐transfer, whereas those receiving CD49a^−^CD8^+^ T cells showed minimal mortality (Figure S5C). Notably, grafts from mice transferred with CD49a^+^CD8^+^ T cells displayed more pronounced immune infiltration and fibrosis formation (Figure S5D).

**FIGURE 4:**
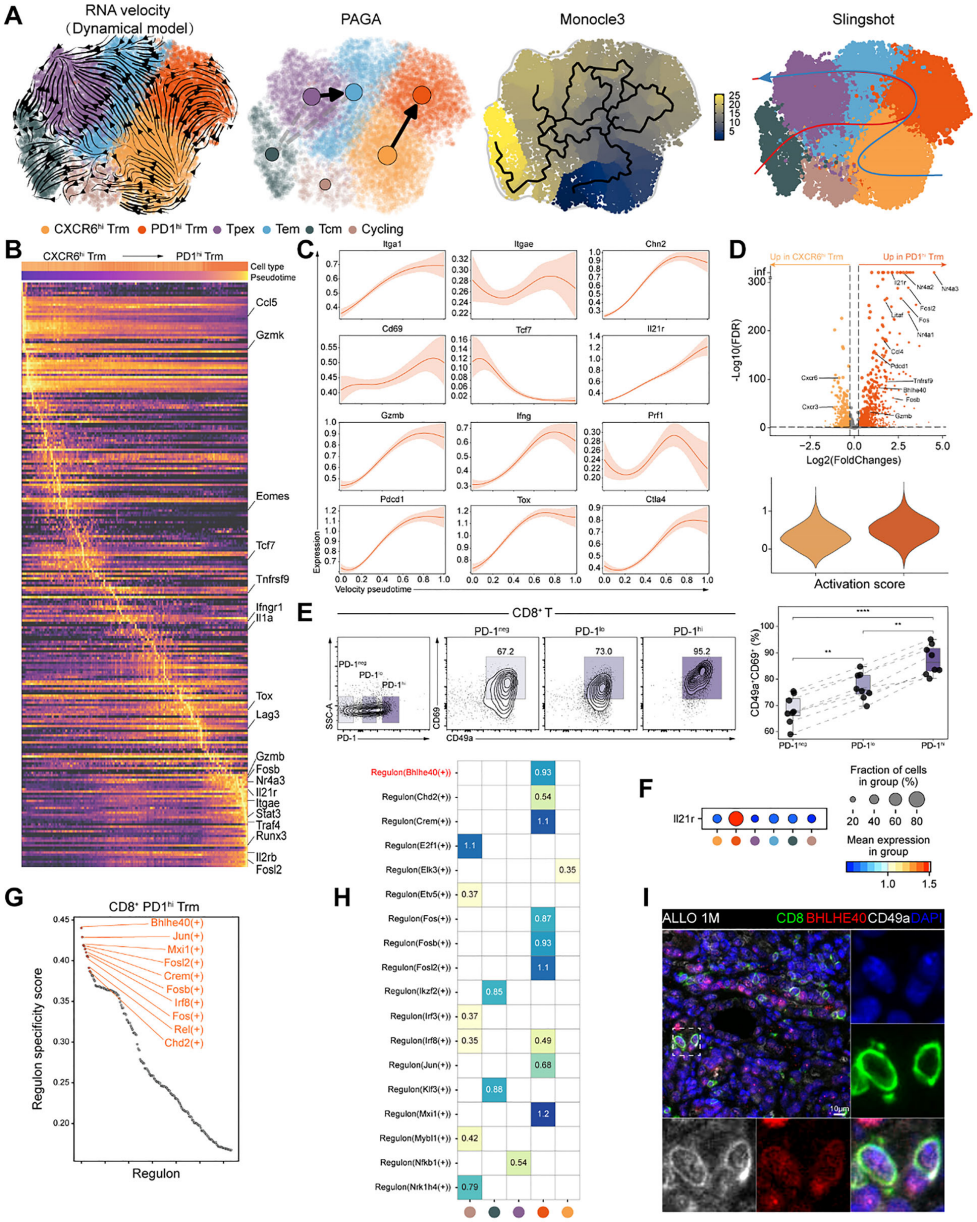
Transition of CD8^+^ T_RM_ cells to Effector Phenotypes in allografts. (A) Differentiation state estimated by RNA velocity, PAGA, monocle3, and slingshot. (B) Heatmap showing genes differentially expressed along the pseudotime trajectory from CXCR6^hi^ to PD1^hi^ T_RM_ cells. (C) Pseudotime expression plots for selected genes along the CXCR6^hi^ to PD1^hi^ T_RM_ trajectory. The *x*‐axis represents velocity pseudotime. (D) Volcano plot of DEGs comparing PD1^hi^ vs CXCR6^hi^ T_RM_ cells (top). Bottom: Violin plot comparing activation scores. (E) CD8^+^ T cells gated on PD‐1 expression (PD‐1^neg^, PD‐1^lo^, and PD‐1^hi^) were analyzed for CD69 and CD49a expression by flow cytometry at 30 days post‐transplantation (*n* = 7–8). (F) Dot plot illustrating the expression level (color) and percentage of expressing cells (dot size) for *Il21r* among CD8^+^ T cell subclusters. (G,H) pySCENIC analysis of key regulons. RSS ranking of specific regulons in CD8^+^ PD1^hi^ T_RM_ cells (G). Heatmap of regulon activity (AUCell scores) across CD8^+^ T cell subclusters (H). (I) Representative immunofluorescence images of CD8, BHLHE40, and CD49a staining in graft sections. Scale bars, 10 µm. Data are mean ± s.e.m. of biologically independent samples. *p*‐values are from a two‐tailed unpaired Student's *t*‐test (E).

T_RM_ cells have traditionally been viewed as a highly differentiated and tissue‐retentive population with notable plasticity. Yet, their diverse maturation states within allografts are not well defined. To investigate the dynamic trajectories of T_RM_ cell differentiation, we employed a vector field‐based deep learning approach to model lineage progression and branching. We observed a differentiation trajectory from CXCR6^hi^ T_RM_ cells toward PD1^hi^ T_RM_ cells, with higher pseudotime values associated with the latter population (Figure 4A). This pattern was consistently supported by multiple trajectory inference tools, including RNA velocity, Monocle3, PAGA, and Slingshot (Figure 4A) [36, 37, 38]. Heatmaps and gene expression trend plots indicated that adhesion molecules (*Itga1*, *Itgae*), effector molecules (*Gzmb*, *Ifng*), and exhaustion markers (*Pdcd1*, *Tox*) were progressively upregulated along the trajectory from CXCR6^hi^ to PD1^hi^ T_RM_ cells, whereas proliferation marker (*Tcf7*) was downregulated (Figure 4B,C), suggesting that CXCR6^hi^ T_RM_ cells function as precursor, but PD1^hi^ T_RM_ cells are effectors. These findings were further supported by volcano plots and gene set enrichment scoring (Figure 4D). Flow cytometry analysis also demonstrated that the proportion of T_RM_ cells increased in parallel with PD1 expression on CD8^+^T cells (Figure 4E). Collectively, these trajectories and phenotypic analyses indicate a differentiation path of T_RM_ cells toward a PD1^hi^ state, which is associated with upregulated effector and exhaustion‐associated gene signatures. Upregulation of IL‐21R signaling may serve as a compensatory mechanism that facilitates their long‐term persistence in the graft (Figure 4F).

### 2.6 Bhlhe40 Programs Tissue Residency in Allograft‐Infiltrating T Cells

To elucidate the regulatory mechanisms governing the differentiation and function of T_RM_ cells, we employed pySCENIC, a computational pipeline designed for single‐cell regulon analysis, to systematically screen transcription factors (TFs) driving specific cell identities [39]. pySCENIC analysis revealed increased regulon activity of *Bhlhe40*, *Jun*, and *Fosl2* in PD1^hi^ T_RM_ cells, suggesting that these transcription factors may contribute to the acquisition of effector‐like and exhaustion‐related programs (Figure 4G,H). Triple immunofluorescence analysis revealed that BHLHE40 was co‐expressed with CD8 and CD49a in T_RM_ cells (Figure 4I).

**FIGURE 5:**
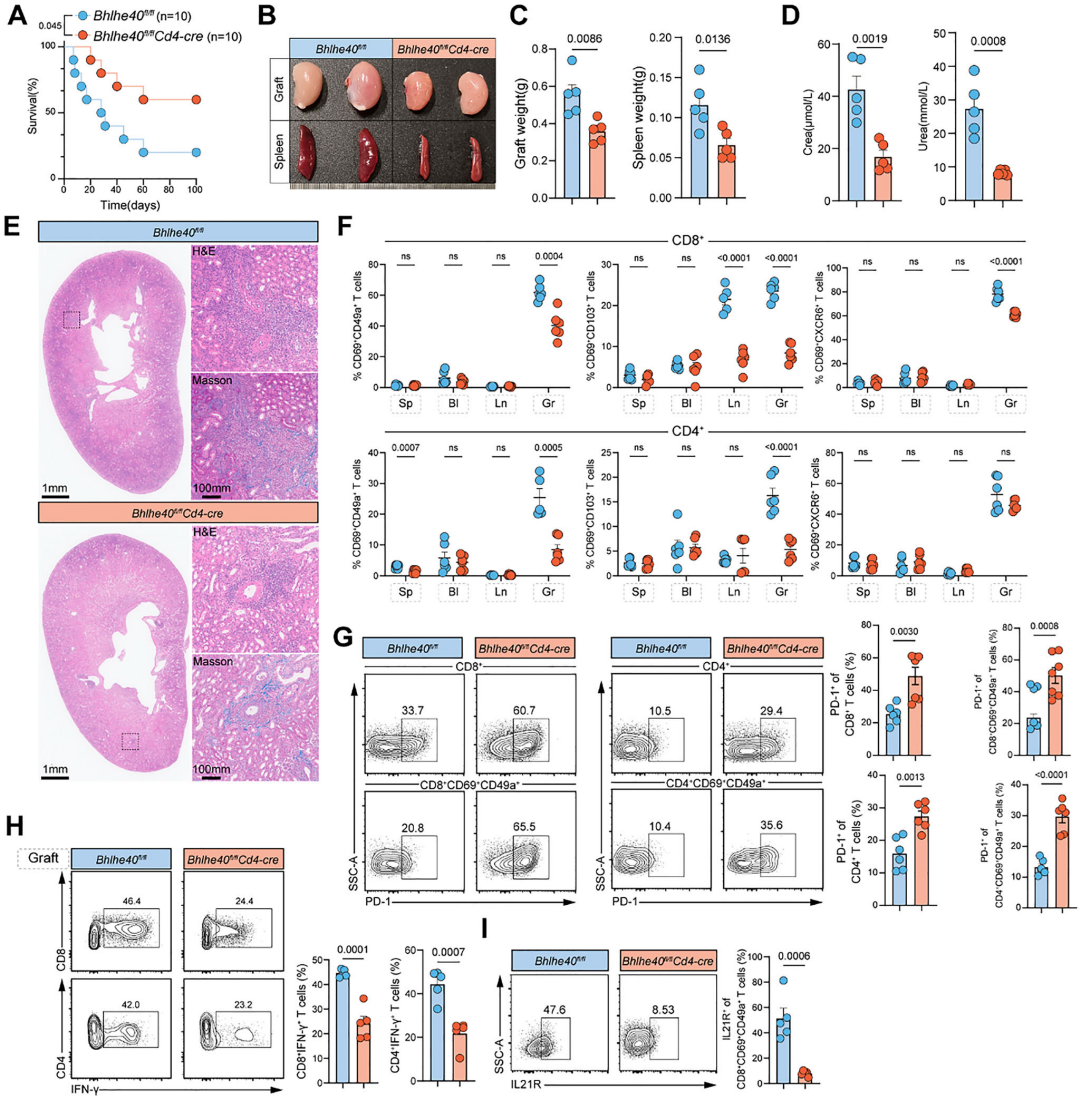
Bhlhe40 programs tissue residency in allograft‐infiltrating T cells. (A) Survival analysis of *Bhlhe40^fl/fl^* (*n* = 10) versus *Bhlhe40^fl/fl^Cd4‐Cre* (*n* = 10) kidney transplant recipients. (B) Representative images of kidneys (top) and spleens (bottom) from *Bhlhe40^fl/fl^* (left) and *Bhlhe40^fl/fl^Cd4‐Cre* (right) mice, harvested 30 days post‐transplant. (C,D) Allograft rejection parameters at day 30 post‐transplant in *Bhlhe40^fl/fl^* (blue) versus *Bhlhe40^fl/fl^Cd4‐Cre* (orange) mice. Measurements include (C) graft and spleen weights, and (D) serum creatinine (Crea) and urea levels (*n* = 5). (E) Representative H&E and Masson's trichrome staining of *Bhlhe40^fl/fl^* and *Bhlhe40^fl/fl^Cd4‐Cre* grafts. (F,I) Frequencies of tissue‐resident T cell subsets in *Bhlhe40^fl/fl^* and *Bhlhe40^fl/fl^ Cd4‐Cre* (*n* = 5–6) mice across spleens, blood, lymph nodes, and grafts. (F) Frequencies of CD69^+^CD49a^+^CD8^+^, CD69^+^CD103^+^CD8^+^, CD69^+^CXCR6^+^CD8^+^, CD69^+^CD49a^+^CD4^+^, CD69^+^CD103^+^CD4^+^, and CD69^+^CXCR6^+^CD4^+^ T cells. (G) Frequencies of PD‐1^+^ cells among CD8^+^, CD4^+^, CD69^+^CD49a^+^CD8^+^, and CD69^+^CD49a^+^ CD4^+^ T cells in grafts (*n* = 6). (H) Frequencies of CD8^+^IFN‐γ^+^ and IFN‐γ^+^CD4^+^ T cells in grafts (*n* = 5). (I) Percentage of IL21R^+^ cells within the CD69^+^CD49a^+^CD8^+^ T cell population in grafts (*n* = 5). Data are mean ± s.e.m. of biologically independent samples. *p*‐values are from a two‐tailed unpaired Student's *t*‐test (C,D, and F–I) and log‐rank test (A).

In the immune system, BHLHE40 has been shown to regulate the development and function of various lymphocyte lineages, including T cells, macrophages, and innate lymphoid cells [40, 41, 42, 43]. To assess whether BHLHE40 regulates T_RM_ cell accumulation in the allograft, we generated mice with T cell‐specific deletion of *Bhlhe40* (*Bhlhe40^fl/fl^Cd4‐Cre*) and analyzed their immune profiles in the context of allorejection compared to wild‐type (*Bhlhe40^fl/fl^*) controls (Figure S6A,B). Kidney transplant survival analysis revealed that *Bhlhe40^fl/fl^Cd4‐Cre* mice exhibited significantly improved survival, with more than 50% of recipients surviving beyond 100 days (Figure 5A). These mice also showed reduced graft and spleen weights, along with lower serum creatinine and blood urea nitrogen levels, indicating better renal function (Figure 5B–D). Histological analysis further supported the protective effect of *Bhlhe40* deficiency. H&E staining revealed markedly reduced tissue injury in the *Bhlhe40^fl/fl^Cd4‐Cre* group, while Masson's trichrome staining showed significantly less fibrotic deposition compared to controls (Figure 5E).

To further investigate the cellular mechanisms, we analyzed the composition of immune cells in the spleen, peripheral blood, mesenteric lymph nodes, and kidney grafts at 30 d post‐transplantation. No significant differences in the proportions of CD4^+^ and CD8^+^ T cells among total CD3^+^ T cells were observed across these tissues between the two groups (Figure S6C,D). We next assessed the frequency of tissue‐residency markers, including CD69^+^CD49a^+^, CD69^+^CD103^+^, and CD69^+^CXCR6^+^ subsets on CD4^+^ and CD8^+^ T cells. Significant differences in the frequencies of CD69^+^CD49a^+^ and CD69^+^CD103^+^ populations were observed in graft‐infiltrating CD4^+^ and CD8^+^ T cells (Figure 5F; Figure S6E). Notably, a significant difference in the CD69^+^CXCR6^+^ subset was observed only in CD8^+^ T cells within the graft, but not in other tissues (Figure 5F). We further observed marked upregulation of PD1 expression in the *Bhlhe40^fl/fl^Cd4‐Cre* group, both in total CD4^+^ and CD8^+^ T cells as well as within CD4^+^ and CD8^+^ T_RM_ subsets (Figure 5G). CD4^+^ and CD8^+^ T cells isolated from grafts and spleens at 4 weeks post‐transplantation were stimulated with PMA/ionomycin for 5 h. Flow cytometry analysis showed significantly reduced IFNγ production in the *Bhlhe40^fl/fl^Cd4‐Cre* group compared to *Bhlhe40^fl/fl^* controls (Figure 5H; Figure S6F). The proportion of IL‐21R^+^ cells among CD4^+^ and CD8^+^ T_RM_ cells was also significantly reduced in *Bhlhe40^fl/fl^Cd4‐Cre* group (Figure 5I). In brief, T cell‐specific deletion of *Bhlhe40* profoundly reshaped the differentiation landscape and functional state of graft‐infiltrating T_RM_ cells, leading to reduced effector cytokine production, diminished IL‐21R signaling, and improved allograft survival. These findings establish *Bhlhe40* as a critical transcriptional regulator of tissue residency and functional programming in T_RM_ cells under sustained allogeneic antigenic stimulation.

### 2.7 Transcriptomic and Epigenomic Analyses Identify BHLHE40 as a Key Regulator of T_RM_ Cells

**FIGURE 6:**
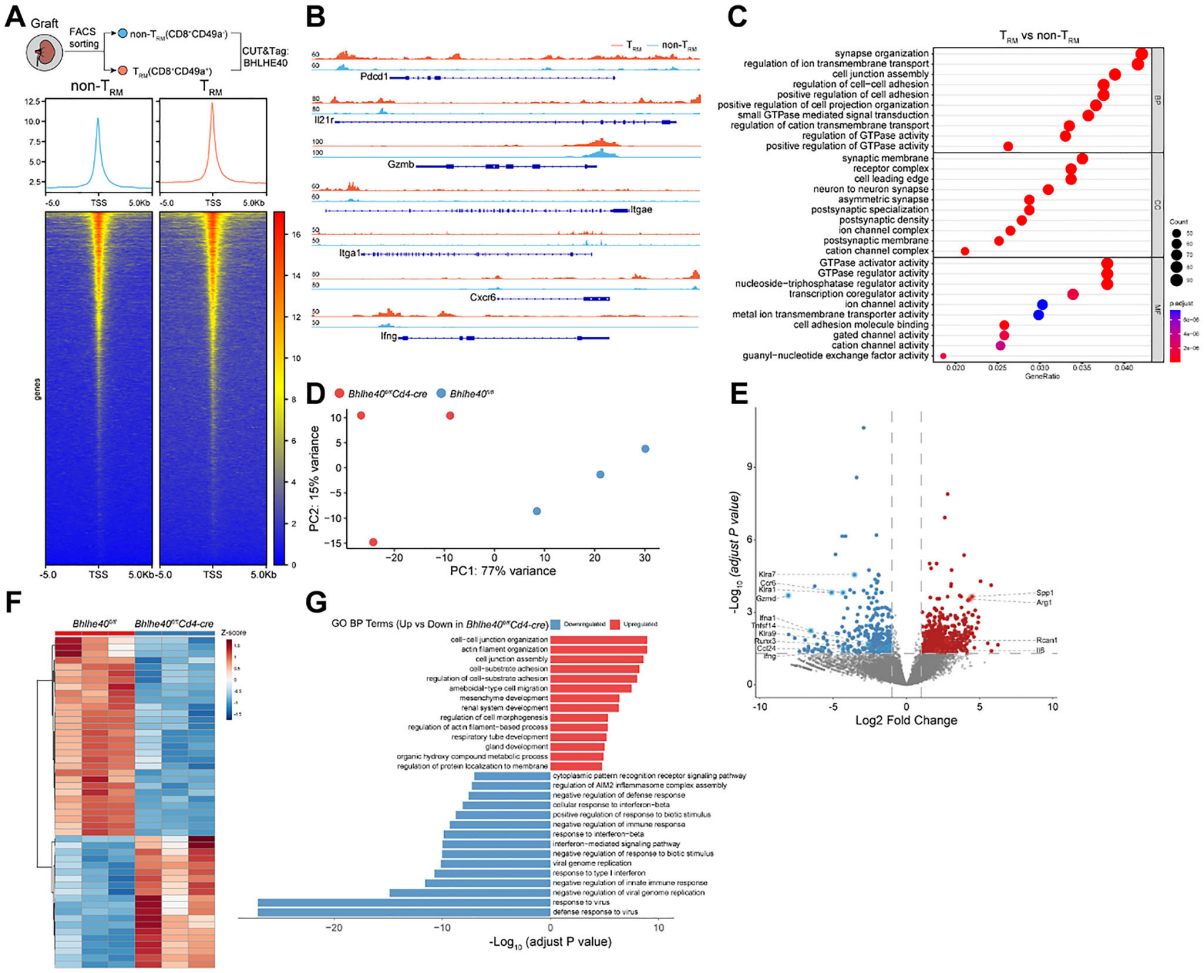
Transcriptomic and epigenomic analyses identify BHLHE40 as a key regulator of T_RM_ cells. (A) Heatmaps and density plot of CUT&Tag signals for BHLHE40 in the transcription start site (TSS) region in sorted graft‐infiltrating CD49a^+^ CD8^+^ or CD49a^−^CD8^+^ cells from grafts. (B) Genome browser views of BHLHE40 CUT&Tag signals at *Pdcd1*, *Il21r*, *Gzmb*, *Itgae*, *Itga1*, *Cxcr6*, and *Ifng* loci. (C) Dot plot showing Gene Ontology (GO) pathway enrichment analysis for potential BHLHE40 target genes, defined as genes upregulated in T_RM_ compared to non‐T_RM_ cells. (D) Principal Component Analysis (PCA) of bulk RNA‐seq data from sorted graft‐infiltrating CD8^+^ T cells comparing *Bhlhe40^fl/fl^* (*n* = 3) and *Bhlhe40^fl/fl^Cd4‐Cre* (*n* = 3) recipients. (E,F) Differential expression analysis of graft‐infiltrating CD8^+^ T cells comparing *Bhlhe40^fl/fl^Cd4‐Cre* (*n* = 3) versus *Bhlhe40^fl/fl^* (*n* = 3). (E) Volcano plot showing DEGs (up: 656, red; down: 291, blue). (F) Heatmap visualizing expression Z‐scores of DEGs across samples. (G) Top enriched Gene Ontology (GO) Biological Process (BP) terms for genes upregulated (red) or downregulated (blue) in *Bhlhe40^fl/fl^Cd4‐Cre* versus *Bhlhe40^fl/fl^*.

To dissect the transcriptional role of Bhlhe40 in the transplant microenvironment, we employed CUT&Tag and RNA‐seq on sorted CD49a^+^ CD8^+^ T_RM_ and CD49a^−^CD8^+^ non‐T_RM_ cells from kidney allografts. While BHLHE40 bound broadly near TSSs in both subsets, its binding was significantly enriched at the promoters of key T_RM_‐associated genes (*Itga1*, *Itgae*, *Pdcd1*, *Ifng*) (Figure 6A,B). Pathway analysis of Bhlhe40 binding sites revealed significant enrichment in transcription coregulator activity, synapse organization, and cell junction assembly, specifically in T_RM_ cells compared to non‐T_RM_ cells (Figure 6C).

To assess the functional consequences of Bhlhe40 activity, we performed RNA‐seq on sorted CD8^+^ T cells from grafts of *Bhlhe40^fl/fl^Cd4‐Cre* mice and *Bhlhe40^fl/fl^* controls. *Bhlhe40* deficiency led to profound transcriptomic changes, including significant downregulation of effector genes (*Ifng*, *Gzmb*, *Tnfsf14*) and master regulators of tissue residency (*Runx3*, *Eomes*) (Figure 6D–F). Intriguingly, enrichment analysis highlighted a downregulation of negative immune regulation and interferon response pathways, but an upregulation of pathways linked to ‘local adhesion’ in *Bhlhe40^fl/fl^Cd4‐Cre* CD8^+^ T cells (Figure 6G). This suggests BHLHE40 plays a complex, dual role in balancing T cell activation, suppression, and tissue interaction.

In summary, loss of *Bhlhe40* in CD8^+^ T cells during kidney transplantation results in a complex phenotype characterized by diminished effector capacity, potential signs of exhaustion, hindered T_RM_ development, and altered adhesion/migration properties, ultimately leading to a functionally constrained and partially exhausted state.

### 2.8 TGF‐β Signaling is Required for the Development of T_RM_ Cells in TLSs

**FIGURE 7:**
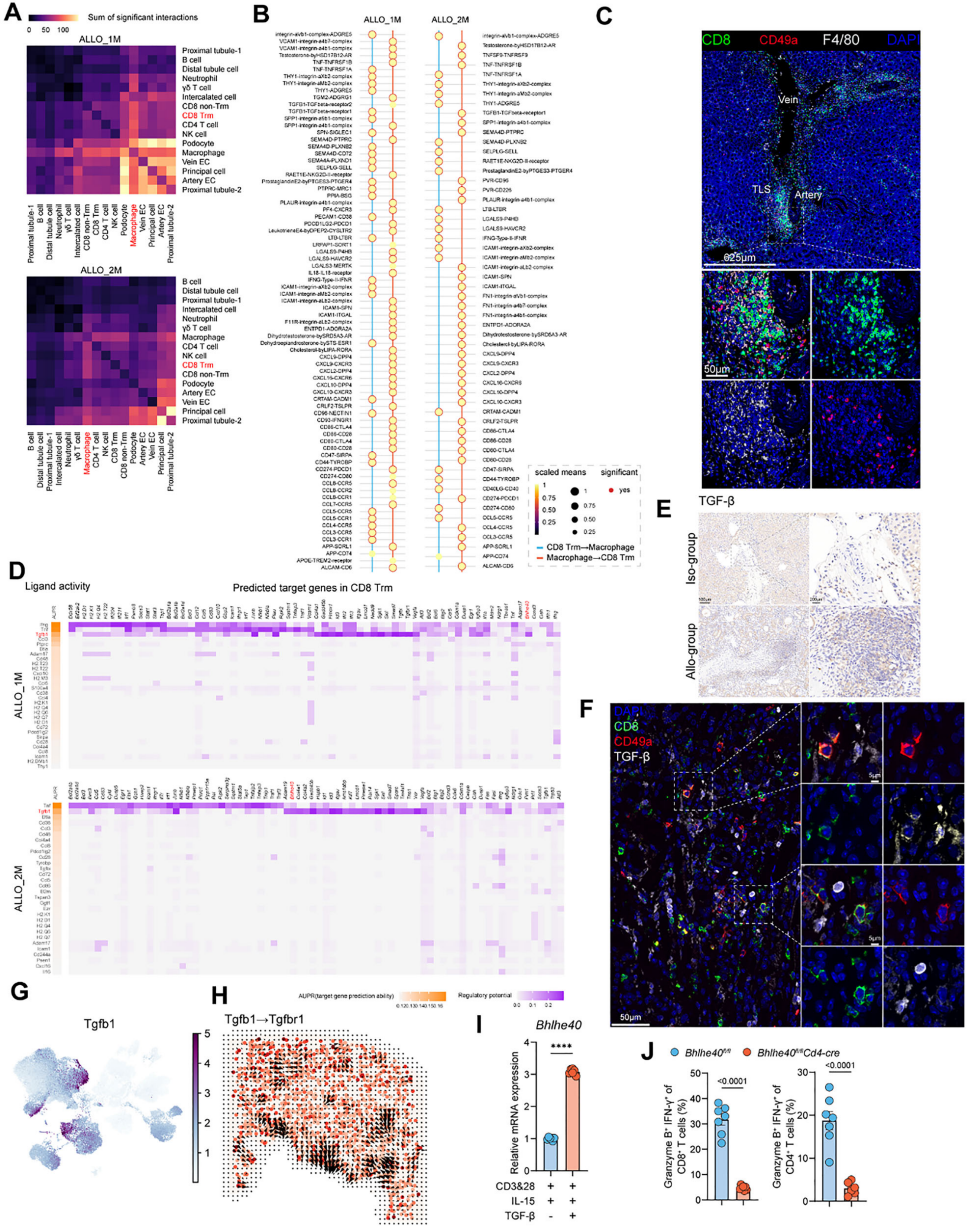
TGF‐β signaling is required for the development of T_RM_ cells in TLSs. (A) Cell‐cell communication analysis of graft‐infiltrating immune and parenchymal populations at one month (Allo_1 m) and two months (Allo_2 m) post‐transplantation, inferred using CellPhoneDB. Heatmaps display the total number of significant ligand–receptor interactions between the indicated cell types. (B) Dot plots showing representative ligand–receptor pairs between CD8^+^ T_RM_ cells and macrophages at one month (Allo_1 m) and two months (Allo_2 m) post‐transplantation. (C) Representative immunofluorescence images of CD8, F4/80 and CD49a staining in graft sections at day30 post‐transplantation. Scale bars, 625 and 50 µm. (D) Heatmaps showing the predicted regulatory potential of top‐ranked ligands on target genes in CD8 T_RM_ cells using NicheNet analysis. (E) The grafts from Iso‐group and Allo‐group mice were subjected to immunohistochemical staining for TGF‐β. Scale bars, 100 and 200 µm. (F) Representative immunofluorescence images of CD8, TGF‐β and CD49a staining in graft sections at day30 post‐transplantation. Scale bars, 50 and 5 µm. (G) UMAP visualization of *Tgfb1* expression across all cells. (H) Spatially resolved inference of *Tgfb1* → *Tgfbr1* signaling interactions based on commot analysis. Arrows indicate the direction and strength of putative ligand–receptor communication between spatially defined sender and receiver cell populations in graft tissue sections. (I) Murine T cells were cultured with or without TGF‐β for 7 days, and *Bhlhe40* expression was measured by qPCR. (J) Expression levels of IFN‐γ and Granzyme B were quantified in CD8^+^ and CD4^+^ T cells after 7‐day culture in the presence of TGF‐β.

To investigate cell–cell interactions in allorejection models, we employed CellPhoneDB to evaluate the relative frequency of interactions among different cell types [44]. We found that in both the ALLO_1 m and ALLO_2 m groups, macrophages exhibited the strongest interactions with CD8^+^ T_RM_ cells, with multiple ligand–receptor pairs showing significant differences (Figure 7A,B). Consistent with these findings described above, immunofluorescence analysis revealed frequent colocalization of CD49a^+^CD8^+^ T cells and F4/80^+^ cells within TLSs (Figure 7C). Additionally, CD49a on CD8^+^ T_RM_ cells was predicted to interact with several collagen‐binding ligands, such as *Col18a1*, *Col4a1*, *Col4a2*, and *Sema7a*, expressed by arterial and venous endothelial cells (Figure S7A,B). These ligands were markedly upregulated in the endothelial compartment of allografts compared to isografts (Figure S7C).

To investigate the key signaling pathways through which the immune microenvironment drives CD8^+^ T_RM_ cells formation, we utilized NicheNet to predict the mediating ligand‐receptor interactions and their downstream target genes [45]. Intriguingly, despite differences in the overall ligand activity profiles between the two time points, transforming growth factor beta 1 (*Tgfb1*) consistently emerged as a highly ranked ligand predicted to regulate the transcription factor *Bhlhe40* in CD8^+^ T_RM_ cells in both ALLO_1 m and ALLO_2 m groups (Figure 7D).  Both immunohistochemical and immunofluorescent staining demonstrated high levels of TGF‐β in tertiary lymphoid structures (TLSs) and surrounding CD8^+^ T_RM_ cells (Figure 7E,F). UMAP plots revealed high expression of *Tgfb1* in CD4^+^ T cells, NK cells, and macrophages (Figure 7G). Interestingly, spatial communication analysis using *commot* identified a *Tgfb1→Tgfbr1* signaling axis specifically localized within the previously defined TLS regions (Figure 7H) [46]. Given our prediction that TGF‐β signaling promotes the expression of the transcription factor *Bhlhe40* in CD8^+^ T_RM_ cells, we next asked whether TGF‐β stimulation could promote the phenotypic maturation of CD8^+^ T cells into T_RM_‐like cells in vitro. We initially confirmed that TGF‐β treatment elevated *Bhlhe40* expression in T cells (Figure 7I). Subsequently, assessing the T_RM_‐like phenotype marked by CD49a expression, we found that T cells (both CD8^+^ and CD4^+^) from *Bhlhe40^fl/fl^Cd4‐Cre* mice exhibited an impaired capacity to acquire this resident phenotype upon TGF‐β stimulation, relative to *Bhlhe40^fl/fl^* T cells (Figure S7D). Consistent with this impaired differentiation, and despite similar proliferation rates between the groups upon TGF‐β treatment, *Bhlhe40^fl/fl^Cd4‐Cre* T cells also displayed markedly lower levels of the effector molecules Granzyme B and Interferon‐gamma (Figure 7J; Figure S7E,F). Thus, our combined computational, spatial, and functional analyses converge to identify the TGF‐β‐Bhlhe40 signaling axis as a key regulatory mechanism governing CD8^+^ T_RM_ cells development within the specific microenvironment of allograft TLSs.

### 2.9 Identification of T_RM_ Cell Populations in Human Rejecting Allografts

**FIGURE 8:**
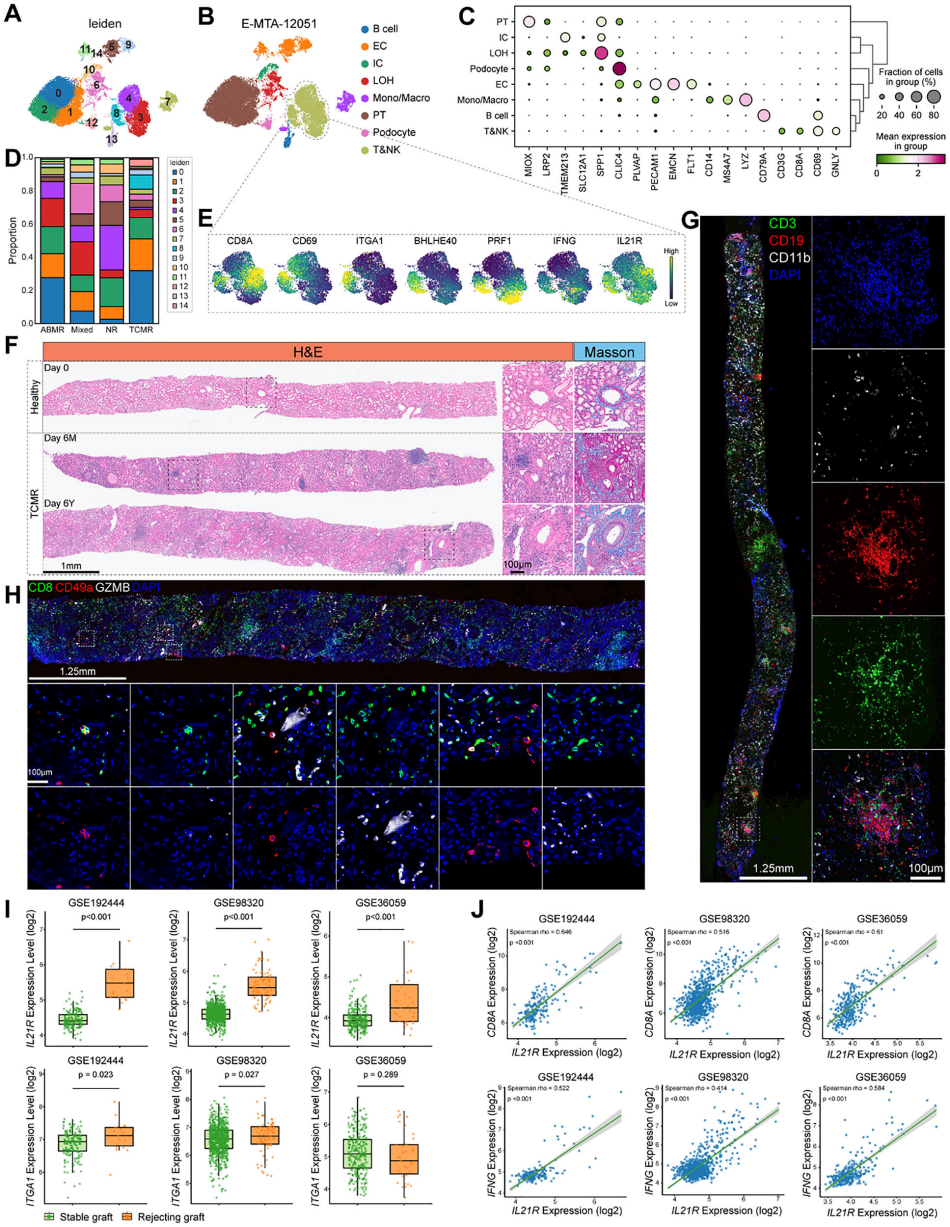
Identification of T_RM_ cell populations in human rejecting allografts. (A–E) Analysis of a public single‐cell RNA‐sequencing dataset (E‐MTAB‐12051) derived from 16 human kidney transplant biopsies representing diverse clinical phenotypes: No Rejection (NR), Mixed Rejection, Antibody‐Mediated Rejection (ABMR), and T‐Cell Mediated Rejection (TCMR). UMAP visualizations of integrated cells colored by cell cluster identity (A, B). Dot plot showing expression patterns (average expression, color; percent expressed, dot size) of selected marker genes across clusters (C). Compositional analysis showing the proportion of each cell cluster within each phenotype group (D). UMAP feature plots showing expression of selected key genes including *CD8A*, *CD69*, *ITGA1*, *BHLHE40*, *PRF1*, *IFNG*, and *IL21R* in T&NK cells (E). (F) Representative H&E and Masson's trichrome staining of control (healthy donor) kidney tissue and TCMR grafts (at 6 months and 6 years post‐transplant). Scale bars, 1 mm and 100 µm. (G,H) Representative immunofluorescence images of CD3, CD19, CD11b (G), CD8, CD49a, and GZMB (H) staining in graft sections. Scale bars, 1.25 mm and 100 µm. (I,J) Validation using public bulk RNA‐seq datasets (GSE19244, GSE98320, and GSE36059) of human kidney allografts. Box plots comparing log2 expression levels of *IL21R* and *ITGA1* between stable (green) and rejecting (orange) grafts (I). Statistical significance between stable and rejecting grafts for each gene was determined using a two‐sided Wilcoxon rank‐sum test. *p*‐values are indicated on the plots. Scatter plots demonstrating the positive correlation between log2 expression of *IL21R* and *CD8A* or *IFNG*. Spearman correlation coefficient (rho) and *p*‐value are shown, along with linear regression lines (green) and 95% confidence intervals (J).

Having established the presence and functional significance of CD49a^+^CD8^+^ T_RM_ cells in mouse kidney allografts, we next interrogated human data to explore their correlates in clinical transplantation. To corroborate our findings in human kidney allografts, we re‐analyzed a publicly available scRNA‐seq dataset (E‐MTAB‐12051) comprising 16 renal allograft biopsies spanning a range of rejection severities (Figure 8A) [47]. This analysis identified eight transcriptionally 8 cell clusters (Figure 8B,C). Notably, within the T and NK cell populations, we identified a specific cluster (Leiden 8) highly expressing key genes associated with CD8^+^ T_RM_ identity and function, including *CD8A, CD69, ITGA1, BHLHE40, PRF1, IFNG*, and *IL21R* (Figure 8E). Analysis of individual biopsies revealed that this cluster was notably prominent in specific grafts undergoing T cell‐mediated rejection (TCMR) (e.g., Sample 16), although considerable heterogeneity was observed across samples (Figure 8D; Figure S8A). This variability likely reflects the focal distribution of immune infiltrates (such as TLSs) and the inherent sampling bias associated with clinical core needle biopsies.

To validate these findings in a clinical context, we examined an independent cohort of human kidney allograft biopsies collected from our center. Histological examination of human kidney allograft biopsies confirmed hallmark features of TCMR, including intense immune infiltration, pronounced fibrosis, and the presence of well‐formed TLSs compared to healthy controls (Figure 8F,G). Immunostaining further revealed frequent co‐localization of CD8^+^, CD49a^+^, and GZMB^+^ cells within the rejecting tissues (Figure 8H).

Given that *IL21R* expression was largely restricted to immune cells in the scRNA‐seq analysis, unlike *ITGA1*, which was also detected in parenchymal cells, we hypothesized that *IL21R* might serve as a more specific marker for effector‐like T_RM_ cells. To test this, we analyzed bulk RNA‐seq data from three independent kidney transplant cohorts (GSE19244, GSE98320, GSE36059) [48, 49, 50]. Consistent with the presence of the activated T_RM_ cells, key genes including *CD8A*, CXCR6, *GZMB*, *IFNG*, *IL21R*, *ITGA1*, and *ITGAE* were significantly upregulated in rejecting grafts relative to stable grafts (Figure 8I; Figure S8B). Moreover, supporting its specific association with cytotoxic effector function, *IL21R* expression positively correlated with *CD8A*, *IFNG*, and *GZMB* expression across these datasets (Figure 8J; Figure S8C). Taken together, these findings identify a cytotoxic CD8^+^ T_RM_ cell population enriched in rejecting human allografts and highlight *IL21R* as a candidate marker for this effector subset.

## 3 Discussion

By integrating single‐cell, spatial transcriptomic, and functional analyses, we reveal that chronic kidney allograft rejection fosters a unique immunological milieu conducive to the development and persistence of T_RM_ cell subsets, distinct from the dynamics of acute rejection. Our study identified a CD49a^+^CD8^+^ T_RM_ cell population in chronically rejecting grafts 2 months after transplantation. These T_RM_ were sufficient to mediate rejection independently. These CD8^+^ T_RM_ cells progressively mature from a CXCR6^hi^ precursor‐like state to a CD49a^+^PD1^hi^ effector phenotype, characterized by enhanced cytotoxicity (*Gzmb*, *Ifng*) and upregulation of exhaustion markers (*Pdcd1*, *Tox*). These CD49a^+^PD1^hi^ T_RM_ produces more GZMB and IFN‐γ, which is different from exhausted T cells in tumor and infection. Notably, we identified the transcription factor BHLHE40 as the core regulator of these effector phenotype T_RM_ cells and further demonstrated that BHLHE40 govern CD8^+^ T_RM_ cell differentiation, accumulation, and effector function, thereby driving chronic rejection in a murine kidney allograft model. BHLHE40 deficiency attenuated T_RM_ cell infiltration and prolonged allograft survival, underscoring its pivotal pathogenic role. These findings shift the transplant rejection paradigm by highlighting a BHLHE40‐orchestrated, resident immune component, rather than circulating cells, as a major contributor to chronic allograft injury.

The heterogeneity of T_RM_ cells is profoundly shaped by factors such as the tissue microenvironment [51, 52, 53, 54], the nature and duration of antigen exposure [20, 55], cytokine signaling [21, 56], transcriptional and epigenetic regulation [11, 57, 58, 59, 60], as well as interactions between stromal and immune cells [61]. Therefore, precisely defining T_RM_ subsets within specific tissues and disease contexts is of critical importance. While CD69 and CD103 are predominantly utilized as residency markers in most studies, their expression levels do not always perfectly correlate with actual tissue retention capabilities [11, 62, 63]. Given the absence of a universal residence‐specific signature, our study first identified, through differential gene screening and flow cytometric analysis, that CD49a serves as a superior residency marker compared to CD103 and CXCR6 in our context. Notably, CD49a also appears to mediate T_RM_ cell interaction with collagen ligands within the graft, thereby potentially enhancing their resident phenotype. Furthermore, we uncovered significant heterogeneity within the CD8^+^ T_RM_ compartment, distinguishing two prominent populations. These cells exhibit a progressive differentiation toward an effector state, characterized by high expression of IFN‐γ, GZMB, and PD‐1, with this effector‐resident subset also displaying elevated levels of IL21R. These effector T_RM_ cells were derived from CXCR6^hi^ T_RM_ with a precursor‐like state at a late stage (2 months). Interestingly, our data showed that precursor CXCR6^hi^ T_RM_ matured from naive T cells at an early stage (<1 month). These findings unravel a program that precursor CXCR6^hi^ T_RM_ developed to effector CD49a^+^PD1^hi^ CD8^+^ T_RM_, different from T_RM_ in tumor tissues. This difference might owe to two reasons. First, it is short term observation using tumor bearing mouse model. Second, it is different between the TCR‐self MHC setting and TCR‐non self MHC setting.

The tissue residency of CD8^+^ T_RM_ cells in rejecting allografts underscores the pivotal role of local cues within the graft microenvironment for their maintenance and function. Our spatial transcriptomics and imaging analyses identified TLSs as key niches where T_RM_ cells cluster and likely receive instructive signals, notably a pronounced TGF‐β signaling signature. This aligns with prior work implicating TGF‐β in T_RM_ cells differentiation [11, 53, 64], and our data significantly extend these findings by positioning BHLHE40 downstream of TGF‐β as a transcriptional integrator of pro‐residency and effector programs. This delineated TGF‐β‐BHLHE40 axis, operating within these allograft‐associated TLSs, is thus essential for T_RM_ cells induction and programming. The established role of this axis in coordinating T_RM_ cells retention and pathogenic activation makes it a compelling therapeutic target. This nuanced understanding suggests that spatially or temporally constrained modulation of TGF‐β signaling, rather than broad inhibition, could selectively abrogate pathogenic T_RM_ cell formation while preserving its systemic beneficial functions, offering a translatable therapeutic strategy.

Mechanistically, BHLHE40, activated by upstream signals like TGF‐β, orchestrates a transcriptional network promoting T_RM_ cells effector function and tissue retention. Our transcriptomic and epigenomic analyses reveal BHLHE40 binding and potential chromatin accessibility enhancement at loci for cytotoxic mediators (*Gzmb*, *Ifng*) and adhesion molecules (*Itgae*), alongside suppression of pro‐apoptotic genes, thereby fostering T_RM_ cell persistence. While these multifaceted functions are reminiscent of BHLHE40's roles in other tissue‐resident lymphocytes [65, 66], they appear uniquely co‐opted in alloimmunity, providing a comprehensive view of the transcriptional circuitry sustaining pathogenic T_RM_ responses in allograft rejection. As a central transcriptional checkpoint, BHLHE40 thus represents a compelling therapeutic target.

IL‐21 signaling is recognized as a key molecular pathway driving enhanced T cell cytotoxicity while sustaining cellular function and preventing complete exhaustion under chronic antigen stimulation [67, 68, 69]. Similarly, our study demonstrates that under persistent alloantigen exposure, IL21R expression marks a T_RM_ subset with heightened effector potential and cytotoxicity. Furthermore, IL21R was identified as a selective marker for effector T_RM_ cells in both murine and human rejecting allografts. The enrichment of IL21R^+^ T_RM_ cells in human rejection biopsies suggests cross‐species conservation of this pathogenic subset, highlighting IL21R's potential as both a biomarker and a therapeutic entry point. This raises the possibility that targeting IL‐21 signaling, alone or combined with BHLHE40 inhibition, could selectively modulate deleterious T_RM_ responses while sparing other immune compartments, representing a potential paradigm shift in transplant therapeutics. Nevertheless, the human biopsy data included here are limited in scope, and future studies with larger cohorts and functional validation will be required to confirm the translational relevance of these findings.

In conclusion, this study reshapes our understanding of chronic kidney allograft rejection by positioning BHLHE40 as a central regulator of CD8^+^ T_RM_ cell pathogenicity. We have identified a TGF‐β–BHLHE40 signaling axis operating within the graft microenvironment, particularly in TLSs, that drives T_RM_ cell differentiation and effector function. These insights not only provide a deeper mechanistic understanding of transplant immunobiology but also pave the way for novel therapeutic strategies aimed at selectively targeting these detrimental resident immune cells to promote long‐term allograft acceptance.

## 4 Methods

More detailed information is provided in the Supporting Information.

### 4.1 Study Design

This study aimed to elucidate the formation, function, spatial distribution characteristics, and transcriptional regulation features of alloreactive tissue‐resident memory T cells after mouse kidney transplantation. We first established a mouse kidney transplantation model, in which the recipient's kidneys were removed during the procedure. To explore the characteristics of tissue‐resident memory T cells in the transplanted kidney, we employed a combination of flow cytometry, scRNA‐seq, spatial transcriptomics, CUT&Tag, multiplex immunofluorescence, flow sorting, adoptive transfer, and bulk RNA‐seq techniques. Additionally, we used T cell‐specific Bhlhe40‐knockout mice to investigate the impact of the transcription factor BHLHE40 on T cell differentiation and transplant rejection. Finally, we validated our findings using publicly available databases and patient biopsy specimens.

### 4.2 Statistical Analysis

Statistical analyses were performed using Prism (v10, GraphPad Software) for non‐omics biological data, and R (v4.4.1) or Python (v3.9) for omics‐related analyses. For non‐omics experiments, comparisons were performed using a two‐tailed unpaired Student's *t*‐test for normally distributed data and a log‐rank test for survival analysis, unless otherwise indicated. No outliers were excluded unless explicitly stated. For omics datasets, including scRNA‐seq and bulk RNA‐seq, standard bioinformatic pipelines were employed for analyses such as cell clustering, differential gene expression, cell–cell communication inference (CellPhoneDB, NicheNet, Commot), and correlation analysis. Statistical methods inherent to the relevant software tools (e.g., Wilcoxon rank‐sum test, permutation testing, Pearson or Spearman correlation in Seurat or Scanpy) were used, as detailed in the figure legends. Sample sizes for in vivo experiments were determined based on prior published studies. For in vitro experiments, at least three biologically independent replicates were performed. Unless otherwise indicated, data are presented as mean ± SEM. Statistical significance was defined as *p* ≤ 0.05. Specific *p*‐values and the statistical tests used are provided in the corresponding figure legends.

## Author Contributions

J.L. and B.Y. contributed equally to this work. Z.C. and P.L. designed the research studies. J.L., B.Y., T.P., X.Z., and Z.M. performed most of the experiments. T.P. and D.T. performed in vivo experiments and RNA‐seq analyses. B.X. and J.L. helped with in vivo experiments. J.L. analyzed the data and wrote the manuscript. Z.C. and P.L. supervised the project and provided funding.

## Conflicts of Interest

The authors declare no conflicts of interest.

## Supporting information




**Supporting File**: advs73604‐sup‐0001‐SuppMat.docx

**Supplementary Figures**: advs73604‐sup‐0002‐Figures.docx.

## Data Availability

The data that support the findings of this study are openly available in “the Genome Sequence Archive (GSA)” at “https://ngdc.cncb.ac.cn/gsub,” reference number “26317.”
