# Supplementary material for: BHLHE40 Orchestrates Effector Tissue‐Resident Memory CD8+ T Cells and Limits Long‐Term Survival of Kidney Graft
Source: Adv Sci (Weinh). 2026 Jan 4;13(10):e20518. doi: 10.1002/advs.202520518 (PMC12915110; doi:10.1002/advs.202520518)
Supplement: Supplementary file 1 — Supporting File: advs73604‐sup‐0001‐SuppMat.docx [file ADVS-13-e20518-s001.docx]

**METHODS**

**Mice**

C57BL/6 (B6; H-2^b^) and BALB/c (H-2^d^) mice were purchased from Beijing Vital River Laboratory Animal Technology Co. Ltd (Beijing, China). B6-Rag1-KO(T004753) mice were purchased from GemPharmatech (Nanjing, China). *Bhlhe40^fl/fl^* mice (C57BL/6Smoc-Bhlhe40^em1(flox)Smoc^) were developed by Shanghai Model Organisms Center Inc. *Bhlhe40^fl/fl^* mice were crossed with *Cd4-Cre* mice to generate T cell-specific conditional knockout of *Bhlhe40*. All mice were housed under specific pathogen-free conditions at the Tongji Medical School Facilities for Animal Care and Housing. In this study, all animal protocols were approved by the Institutional Animal Care and Use Committee of Tongji Medical College.

**Mouse kidney transplants**

BALB/c (H-2^d^) mice were used as donors, and C57BL/6 (B6; H-2^b^), *Rag1^-/-^*, or *Bhlhe40^fl/fl^Cd4-Cre* mice were used as recipients, with matched sex for both donors and recipients. The donor’s left kidney, blood vessels, and ureter were collected and placed in HTK preservation solution. First, all native kidneys of the recipient were removed, and then the donor kidney’s artery and vein were anastomosed end-to-side to the recipient’s aorta and inferior vena cava, respectively, and the ureter was implanted into the recipient’s bladder. After blood flow was restored, the pulsation at the anastomosis site and blood flow in the kidney and ureter were observed. The total time from kidney harvest to abdominal closure was approximately 40-50 minutes. Isoflurane anesthesia was used throughout the surgery. Postoperative assessment of recipient activity, mental status, urine output, and mortality was used to determine transplant success and allograft rejection.

**Human clinical sample acquisition**

All patient tissue specimens were collected at the Organ Transplantation Institute of Tongji Hospital. The study protocol was approved by the Ethics Committee of Tongji Hospital, Tongji Medical College, Huazhong University of Science and Technology (Approval No.TJ-IRB202503119). Paraffin-embedded samples were sectioned for H&E, Masson, and multiplex immunohistochemical (mIHC) staining.

**Flow cytometry analysis**

After kidney transplantation, mouse kidney grafts, spleens, mesenteric lymph nodes, and blood were collected. Kidney grafts were dissociated into small pieces using a gentleMACS tissue homogenizer (Miltenyi Biotec) and then incubated in RPMI 1640 medium supplemented with collagenase IV (1 mg/ml, Biofroxx) and DNase I (0.02 mg/ml, biosharp) for 30 minutes at 37°C. The resulting single-cell suspension was filtered through a 70 μm filter and washed by centrifugation at 500 × g for 5 minutes. Spleens and lymph nodes were mechanically dissociated and filtered through a 70 μm filter to obtain single cells. Red blood cell (RBC) lysis was performed on blood and spleen single-cell suspensions using RBC lysis buffer (Solarbio, R1010) for 5 minutes at room temperature. Subsequently, cells were stained for fixable viability dye (Fixable Viability Stain 700, 1:1000, 564997, BD) and cell surface markers and analyzed by flow cytometry (Sony ID7000 or BD FACS Calibur).

For intracellular cytokine staining, cells were stimulated for 5 hours at 37°C with Cell Stimulation Cocktail containing protein transport inhibitors (00-4975-93; eBioscience), followed by fixation and permeabilization using either Cytofix/Cytoperm solution (BD Biosciences) or the FOXP3/Transcription Factor Staining Buffer Set (eBioscience). Cells were then stained with antibodies specific for mouse IFN-γ and Granzyme B.

The following antibodies were used for mouse cell staining: CD45 (1:200, I3/2.3, 752409), CD44 (1:200, IM7, 563970), CD62L (1:200, MEL-14, 563252), CXCR6 (1:300, SA051D1, 151108), PD-1 (1:200, RMP1-30, 748242), IL-21R (1:100, 4A9, 131905), CD8 (1:200, 53-6.7, 562283), CD3 (1:200, 145-2C11, 561825), CD3 (1:200, 17A2, 100236), CD4 (1:200, RM4-4, 116014), CD4 (1:200, GK1.5, 743156), CD69 (1:200, H1.2F3, 741234), CD49a (1:200, Ha31/8, 741549), CD49a (1:200, HMα1, 142609), CD103 (1:200, M290, 741948), IFN-γ (1:200, XMG1.2, 505808), Granzyme B (1:200, GB11, 515403).

**Cell sorting and adoptive transfer of T_RM_ cells**

To obtain tissue-resident memory T cells, kidney grafts were harvested from allo-group B6 recipient mice on postoperative day 30 and processed into single-cell suspensions. Cells were stained at 37 °C for 15 minutes and analyzed by flow cytometry. Live CD45^+^ CD3^+^ CD8^+^ CD49a^+^, CD45^+^ CD3^+^ CD8^+^ CD49a^-^, CD45^+^ CD3^+^ CD49a^+^, and CD45^+^ CD3^+^ CD8^+^ CD49a^+^ cells were sorted by flow cytometry. The sorted cells were then intravenously transferred into *Rag1^-/-^* mice that had received kidney allografts from Balb/c donors. All cell sorting was performed using a FACSAria cell sorter (BD Biosciences).

**Cell culture *in vitro***

CD3^+^ T cells were first isolated from spleen using magnetic-activated cell sorting (MACS), then activated with plate-coated anti-CD3 (5 μg/ml) and anti-CD28 (1 μg/ml) antibodies. Following activation, cells were maintained in complete RPMI 1640 medium supplemented with 10% fetal bovine serum (FBS), 50 μM L-glutamine, 5 mM HEPES, penicillin-streptomycin (10,000 U/ml penicillin and 10,000 μg/ml streptomycin), and 0.05 mM β-mercaptoethanol. The culture medium was further supplemented with IL-2 (10 ng/ml), IL-15 (10 ng/ml), and optionally with TGF-β (10 ng/ml). Cells were cultured at 37°C for 7 days under standard conditions.

**Immunofluorescence and Immunohistochemical Staining**

After harvesting the transplanted mouse kidneys, the tissues were sectioned along the largest surface and fixed overnight in 4% paraformaldehyde. The samples were then processed and embedded in paraffin blocks, and sectioned into 3 μm-thick slices, which were mounted onto glass slides. Unstained matched sections were deparaffinized, rehydrated, and subjected to heat-mediated antigen retrieval in citrate buffer. The sections were then blocked with PBS containing 2% bovine serum albumin (BSA) and 0.05% Tween 20, followed by incubation with primary antibodies at 4°C overnight. The primary antibodies and their dilutions used were as follows: anti-CD3 (for mouse and human; 1:2000, Abcam, ab237721), anti-CD4 (for mouse; 1:1000, Abcam, ab183685), anti-CD8 (for mouse; 1:1000, Abcam, ab209775), anti-GZMB (for mouse and human; 1:3000, Abcam, ab255598), anti-CD49a (for mouse and human; 1:50, LSBio, LS‑C165312), anti-CD103 (for mouse and human; 1:2000, Abcam, ab224202), anti-BHLHE40 (for mouse and human; 1:1000, Novus, NB100-1800), anti-F4/80 (for mouse; 1:500, Cell Signaling Technologies, 70076), anti-PD-1 (for mouse; 1:1000, Abcam, ab214421) and anti-TGF Beta 1 (for mouse and human; 1:1000, proteintech, 21898-1-AP).

**Histopathological analysis**

Sections were stained with hematoxylin-eosin (H&E) or Masson’s trichrome and analyzed using a Leica optical microscope.

**Single-cell RNA-seq data collection**

C57BL/6 (B6) mice underwent kidney transplantation, and at 1 and 2 months post-transplantation, the transplanted kidneys were harvested for further analysis. The kidneys were immediately transferred to cold RPMI-1640 medium (Thermo Fisher) supplemented with 10% fetal bovine serum (FBS, Gibco) and 1% penicillin-streptomycin (Sigma). They were then processed using the gentleMACS Tissue Dissociator (Miltenyi Biotec), which fragmented the tissue into small pieces. The tissue was subsequently incubated in a digestion solution containing collagenase IV (1 mg/ml, Sigma) and DNase I (50 U/ml, Sigma) at 37°C for 30 minutes to achieve complete dissociation into a single-cell suspension. The resulting cell suspension was filtered through a 70-μm cell strainer (Falcon), and viable single cells were counted using Trypan Blue (Thermo Fisher) exclusion. Single-cell suspensions were processed using the 10X Genomics Chromium Controller and the Single Cell 3’ Library and Gel Bead Kit v3.1 (10X Genomics), following the manufacturer’s instructions. Sequencing was performed on an Illumina NovaSeq PE150 platform using 150 bp paired-end reads, targeting a sequencing depth of 30,000 reads per cell.

**Data Preprocessing, Batch Correction, and Clustering**

Sequencing data were processed using Cell Ranger (v6.1.1) aligned to the mm10 mouse reference genome. After initial quality control, the gene expression matrix was processed using Scanpy (v1.10.3). Cells were retained if they met the following criteria: more than 200 detected genes, unique molecular identifier (UMI) counts ranging from 500 to 30,000, and mitochondrial gene expression accounting for less than 15% of the total transcriptome. Genes expressed in fewer than three cells were excluded. For normalization, the data were corrected for library size and log-transformed, ensuring comparability across cells.

To optimize dimensionality reduction and address batch effects, we implemented the single-cell variational inference (scVI) method. First, highly variable genes were selected, and principal component analysis (PCA) was performed to visualize the variance ratio and identify the optimal elbow point, which guided the choice of the *n_latent* parameter for scVI. Batch effects introduced by different 10X library batches ('Sample') were then adjusted using scVI. The scVI model was further optimized by manually tuning the number of layers to improve data integration. In addition, scVI was employed to detect and remove doublets, which are cells that might contain mixed gene expression profiles from multiple cells. This step was crucial for ensuring the accuracy of downstream analyses. The latent representation derived from scVI was used to construct a neighborhood graph, followed by UMAP embedding and Leiden clustering.

Cell type annotation was performed by manually inspecting the expression of highly variable genes across clusters and assigning identities based on known marker genes. Differential gene expression was analyzed using Scanpy’s *tl.rank_genes_groups* function, applying the Wilcoxon rank-sum test to determine statistical significance. Finally, visualizations of gene expression, including violin plots, were generated using Scanpy's plotting tools.

**Differential gene expression and functional enrichment analyses**

DEGs between non-T_RM_ and T_RM_ cells within CD8^+^ T cells or CD4^+^ T cells were identified using the *scanpy.tl.rank_genes_groups* function (method = ‘wilcoxon’). DEGs with a |log2(fold change)| ≥ 0.5, an adjusted P-value ≤ 0.05, and a gene expression fraction > 25% were selected for Gene Ontology (GO) term enrichment analysis. The enrichment analysis was performed using the gseapy Python package (version 1.0.4), specifically the gseapy.enrichr function, with the GO_Biological_Process_2023 gene set.

**Gene Signature Scoring**
To evaluate the functional state of T cell subsets, gene signature scores were calculated using the *score_genes* function in Scanpy. This scoring method computes the average expression of a target gene set subtracted by the average expression of a reference set of genes randomly sampled from the same expression bins to control for technical variation. The specific gene signature used to define the activation/cytotoxicity score included *Ifng, Fasl, Tnf, Gzma, Gzmb, Gzmk, Prf1, Eomes, Nkg7, Slamf7, Pdcd1, Ctla4,* and *Ccl4*. Violin plots visualizing these scores across cell clusters were generated using *sc.pl.violin*.

**Cell trajectory inference**

RNA velocity, PAGA, Slingshot, and Monocle3 were employed to perform CD8^+^ T cell trajectory inference. RNA velocity was used to predict the future states of individual cells based on their splicing kinetics, providing insights into the directionality of cellular differentiation. PAGA was utilized to map the connectivity between distinct cell states, constructing a graph that reflects the global structure of cellular transitions. Slingshot, on the other hand, was applied to infer lineage trajectories by identifying branching points and ordering cells along developmental paths. Finally, Monocle3 was used to perform pseudotime analysis, which enabled the ordering of cells along a continuous differentiation axis, helping to track their progression over time. Together, these tools provided a comprehensive framework for understanding the temporal dynamics and lineage relationships of the cells in our study.

**pySCENIC analysis**

For predicting transcription factors (TFs) in CD8^+^ T cells, we used the pyscenic pipeline, which integrates gene co-expression networks with motif enrichment. The expression matrix was first filtered for highly variable genes (HVGs) using scanpy. A gene regulatory network (GRN) was then constructed using GRNBoost2 to calculate co-expression relationships among genes. Transcription factor binding motifs were identified in the promoters of genes within the GRN using JASPAR and TRANSFAC databases. TF activity scores were computed based on co-expression patterns and motif enrichment to infer the activity of each TF.

**Receptor-ligand interaction inference**

To investigate potential interactions between different cell types, including CD8^+^ T_RM_ cells and macrophages, we performed a cell-cell communication analysis using CellPhoneDB, a publicly available repository that catalogs curated receptor-ligand pairs and their interactions. This analysis was carried out using the CellPhoneDB Python package (version 5.0.1). Receptor-ligand interactions were identified based on the expression of receptors in one cell type and the corresponding ligands in another. Only interactions between receptors and ligands expressed in the relevant subclusters were considered to ensure specificity. To further explore ligand-receptor interactions and their downstream gene targets, we utilized NicheNet (v1.1.1), which integrates gene expression data from our dataset with a prior knowledge model of ligand-to-target signaling pathways. Using the signature genes of CD8^+^ T_RM_ subpopulations across different samples as target gene lists, we predicted potential ligands and their downstream gene targets using the *predict_ligand_activities* function. This analysis provides insights into the molecular mechanisms underlying the immune interactions between CD8^+^ T_RM_ cells and macrophages.

**Data analysis for Stereo-seq**

Mouse kidney grafts were harvested at 1 and 2 months post-transplantation, immediately embedded in O.C.T. compound (Yeasen) after snap-freezing in liquid nitrogen-precooled isopentane for 1 min. Tissue sections (10 μm) were cut using a CryoStar NX60 cryostat (Thermo Fisher Scientific) and mounted onto Stereo-seq chips prepared according to the manufacturer’s instructions (BGI, Shenzhen, China). For histological validation, adjacent sections were stained with hematoxylin and eosin (H&E). Slides were incubated at 37 °C for 1 min, followed by fixation in pre-chilled methanol at −20 °C for 30 min. After sequential treatment with isopropanol, hematoxylin, bluing buffer, and eosin (1 min, 7 min, 2 min, and 40 s, respectively), slides were rinsed, dried, and scanned with an SLIDEVIEW VS200 digital slide scanner (Olympus) at ×20 magnification.

Stereo-seq data were generated using the BGI DNBSEQ-Tx platform. Read 1 contained the spatial coordinate identifier (CID, bases 1-25) and molecular identifier (MID, bases 26-35), while read 2 encoded the transcript sequence. CID sequences were mapped to the designed barcode positions with one-base mismatch tolerance to correct for sequencing errors. Reads with low-quality MIDs (≥2 bases < Q10 or containing ‘N’) were filtered. Cleaned reads were aligned to the mouse reference genome (mm10) using STAR. Mapped reads with MAPQ >10 were retained and collapsed by identical CID/MID/gene combinations. The resulting data were binned into pseudo-spots of 50 × 50 DNBs (bin50), approximating the average size of renal cells, to generate spatial transcriptomic matrices for downstream analyses. All data processing steps were performed using the SAW pipeline.

**Spatial domain module identification using Hotspot**

To identify spatially organized transcriptional modules, we applied the Hotspot algorithm (v0.2.0) to bin50-level Stereo-seq data. Briefly, genes with sufficient expression and spatial autocorrelation (based on Moran’s I) were retained for further analysis. A k-nearest neighbor graph (k=6) was constructed based on spatial proximity of bins, and pairwise gene autocorrelation scores were computed to define modules with shared spatial expression patterns. Modules were then refined via clustering of autocorrelation profiles using hierarchical clustering, and module scores were computed by averaging normalized expression of member genes per bin. Functional enrichment analyses for each module were performed using g:Profiler (https://biit.cs.ut.ee/gprofiler/), based on Gene Ontology and KEGG pathway annotations. Modules with consistent anatomical localization and enriched immune or metabolic signatures were prioritized for further biological interpretation.

**Spatial deconvolution using cell2location**

To map cell types to spatial locations in bin50-level Stereo-seq data, we applied the cell2location algorithm (v0.1.4, https://cell2location.readthedocs.io). Briefly, a reference single-cell RNA-seq dataset was preprocessed to define cell type signatures, retaining highly variable genes and normalizing expression counts. The Stereo-seq data (bin50 resolution) was similarly preprocessed, ensuring compatibility with the reference dataset. Cell2location was used to train a hierarchical Bayesian model, integrating the single-cell reference and spatial data to estimate per-bin cell type abundances. The model was configured with default parameters, including a negative binomial distribution for gene expression and a Gaussian prior for cell type proportions. After model convergence, posterior distributions of cell type abundances were extracted for each bin, enabling visualization of cell type spatial distributions. Cell types with distinct anatomical localization were prioritized for downstream biological interpretation, and results were validated against known histological features of the tissue.

**CUT&Tag**

One month post-transplantation, kidney grafts were harvested from recipient mice, and single-cell suspensions were prepared for flow cytometric analysis. CD8^+^ T cells were sorted into non-resident memory (non-T_RM_, CD8^+^CD49a^−^) and resident memory (T_RM_, CD8^+^CD49a^+^) subsets. Sorted cells (approximately 10,000 per subset) were processed for CUT&Tag using the Vazyme Hyperactive Universal CUT&Tag Assay Kit (#TD504) following the manufacturer’s protocol. Briefly, cells were fixed in 0.1% formaldehyde for 10 minutes, permeabilized, and incubated overnight at 4°C with primary antibody (anti-BHLHE40, for mouse and human; 1:100, Novus, NB100-1800). Secondary antibody incubation and pA-Tn5 transposase-mediated tagmentation followed, with libraries prepared and sequenced on an Illumina NovaSeq 6000 platform (2x150 bp, ~20 million reads per sample). Raw reads were quality-filtered using Trimmomatic (v0.39), aligned to the mm10 genome with BWA-MEM (v0.7.17), and peaks were called using MACS2 (v2.2.7.1). Additional analyses, including peak annotation and visualization, were conducted on the Vazyme cloud platform (http://cloud.vazyme.com:83).

**Quantitative RT-PCR**

Total cellular RNA was extracted using the Total RNA Rapid Extraction Kit (Fastagen, Shanghai, China). One microgram of RNA was reverse-transcribed into cDNA using the reverse transcription system (TAKARA, Shiga, Japan). Quantitative real-time PCR (qRT-PCR) was performed on a StepOne Real-Time PCR System (Thermo Fisher Scientific). Gene expression levels were quantified using the comparative Ct (ΔΔCt) method and normalized to GAPDH as an internal control. The primer sequences used were as follows: *Bhlhe40*, forward 5′-CTCCTACCCGAACATCTCAAAC-3′ and reverse 5′-CCAGAACCACTGCTTTTTCC-3′; *Gapdh*, forward 5′-CTGGGCTACACTGAGCACC-3′ and reverse 5′-AAGTGGTCGTTGAGGGCAATG-3′.

**Analysis of bulk RNA-seq data**

On day 30 post-kidney transplantation, CD8^+^ T cells were sorted from kidney grafts of *Bhlhe40^fl/fl^* and *Bhlhe40^fl/fl^Cd4-Cre* recipient mice. Each group (*Bhlhe40^fl/fl^* and *Bhlhe40^fl/fl^Cd4-Cre*) included three biological replicates, and total RNA was extracted using RNAiso Plus (9108; TaKaRa). Briefly, following RNA quality control, cDNA synthesis, end repair, A-tailing, and ligation of Illumina indexed adapters were performed using the TruSeq PE Cluster Kit v3-cBot-HS (Illumina, PE401-3001). Library concentration and fragment distribution were assessed with the Agilent Bioanalyzer 2100 system. Sequencing was conducted on the Illumina NovaSeq 6000 platform, generating paired-end 150 bp reads. Raw reads were quality-filtered using fastp (v0.23.2) to remove adapters and low-quality bases (Phred <20). Filtered reads were aligned to the mouse reference genome (GRCm38/mm10) using STAR (v2.7.6a) with default parameters. Gene expression was quantified using featureCounts (Subread, v2.0.3) to generate raw count matrices based on exon-level reads. Differential expression analysis was performed using edgeR (v3.40.2) in R, applying a quasi-likelihood F-test with Benjamini-Hochberg correction (adjusted p < 0.05, fold change > 2) to identify differentially expressed genes (DEGs) between CD8^+^ T cells from *Bhlhe40^fl/fl^* and *Bhlhe40^fl/fl^Cd4-Cre* mice. Normalized counts were visualized using heatmaps and volcano plots. Functional enrichment analysis of DEGs was conducted using clusterProfiler (v4.4.4) to identify enriched Gene Ontology (GO) terms and KEGG pathways relevant to T-cell function and immune regulation, with a q-value cutoff of 0.05. Gene Set Enrichment Analysis (GSEA, v4.3.2) was performed using MSigDB’s immunological signatures to further explore Bhlhe40-dependent pathways.

**Public dataset analysis**

scRNA-seq fastq files(E_MTAB_12051) from 16 renal allograft needle biopsy tissues, exhibiting diverse phenotypes and rejection severities (ranging from none to severe), were downloaded. Matrix files were regenerated using the Cell Ranger pipeline. Data preprocessing, normalization, integration, and clustering were conducted in Scanpy. Briefly, each sample’s gene expression matrix was filtered by retaining cells with >200 but <5,000 detected genes and genes detected in >3 cells, while cells with >10% mitochondrial gene content were excluded. Matrices from all samples were integrated using the mutual nearest neighbors (MNN) algorithm with 4,002 highly variable genes (HVGs) variable in at least two samples. Clustering of the integrated matrix, performed with the Leiden algorithm, identified 8 clusters, followed by differential gene expression analysis across clusters for annotation.

This study utilized public bulk RNA-sequencing datasets (GSE19244, GSE98320, GSE36059) obtained from the Gene Expression Omnibus (GEO). These datasets were typically pre-processed, for instance, normalized using methods like the trimmed mean of M-values (TMM), with gene expression values subsequently log2 transformed. Based on this normalized expression data, we generated boxplots to compare the expression levels of specific genes between different groups, assessing statistical significance using two-sided Wilcoxon rank-sum test. Furthermore, scatter plots were employed to analyze the correlation between the expression of different genes, and Spearman's rank correlation coefficient (rho) along with its corresponding p-value were calculated. All plots were generated using the ggplot2 package in R.
