# Supplementary material for: BHLHE40 Orchestrates Effector Tissue‐Resident Memory CD8+ T Cells and Limits Long‐Term Survival of Kidney Graft
Source: Adv Sci (Weinh). 2026 Jan 4;13(10):e20518. doi: 10.1002/advs.202520518 (PMC12915110; doi:10.1002/advs.202520518)
Supplement: Supplementary file 2 — Supplementary Figures: advs73604‐sup‐0002‐Figures.docx. [file ADVS-13-e20518-s002.docx]

**
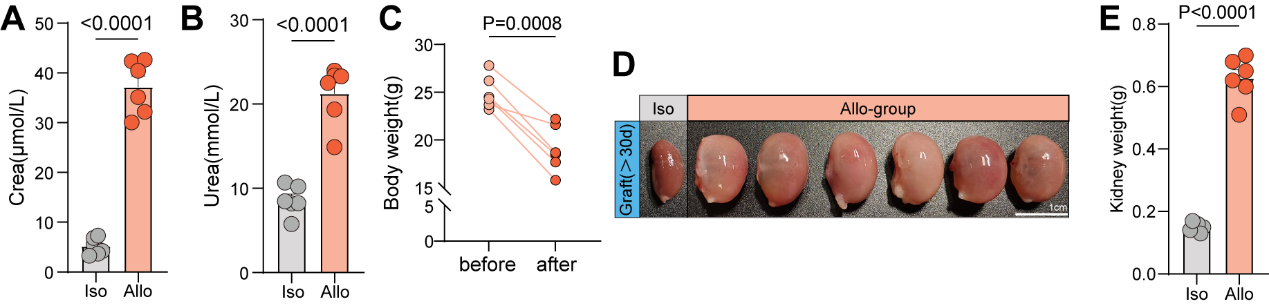
**

**Figure S1. Identification of rejecting phenotypes in murine allogeneic kidney transplantation.**

**A**-**B**, Serum creatinine (Crea) levels (A) and blood urea nitrogen (Urea) levels (B) at day 30 post-transplant (n = 6).

**C**, Body weight of allogeneic kidney transplant recipients pre-transplantation and at day 30 post-transplantation (n = 6).

**D**, Representative gross morphology of kidney isografts and allografts explanted at day 30 post-transplantation. Scale bar, 1 cm.

**E**, Kidney weights of isografts (Iso-group, n = 6) and allografts (Allo-group, n = 6) at day 30 post-transplantation.

Data are mean ± s.e.m. of biologically independent samples. P values are from a two-tailed unpaired Student’s t-test (A, B, C and E).


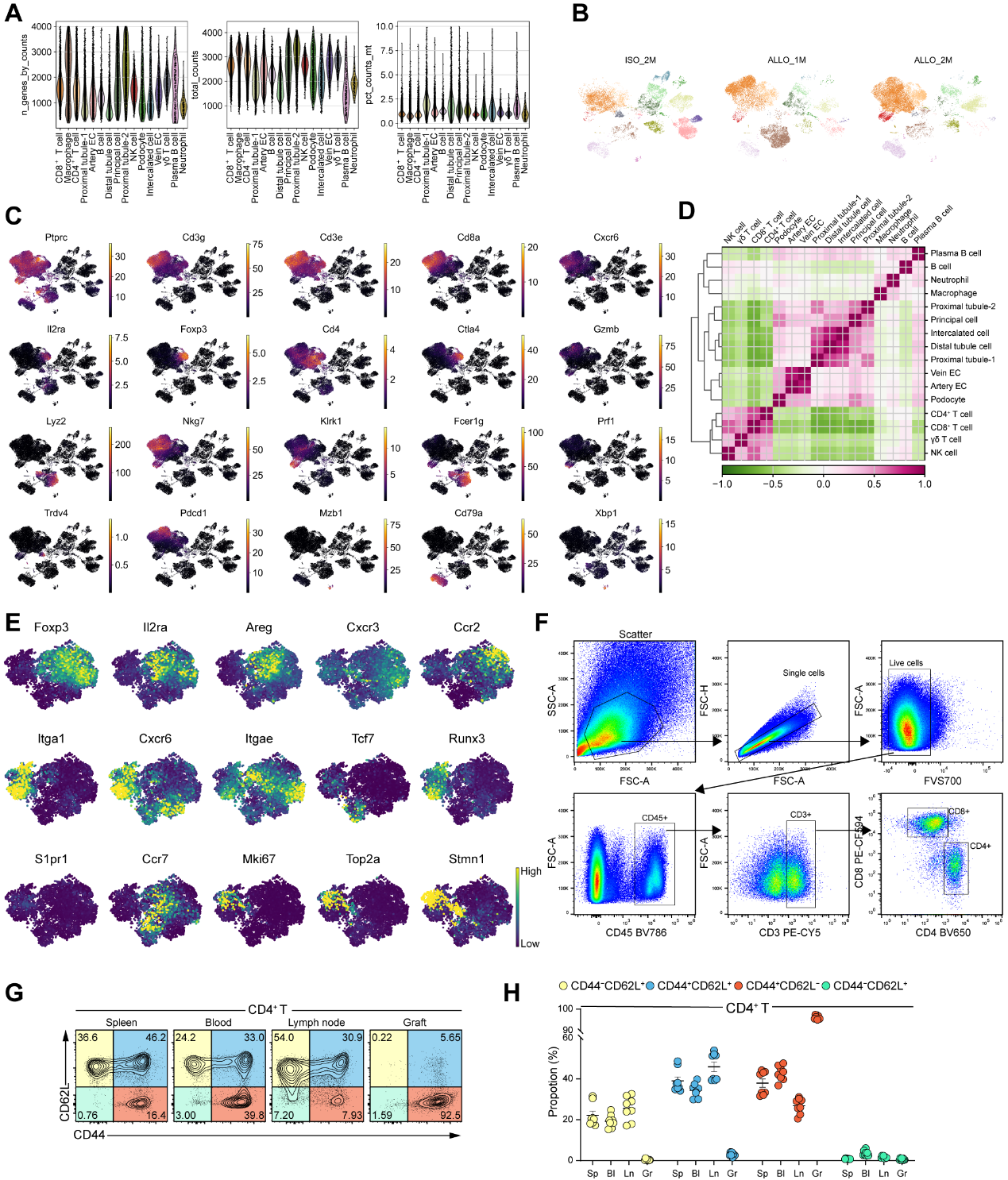


**Figure S2. Single-cell transcriptome analysis and associated gating strategy.**

**A**, Violin plots showing the distribution of the number of detected genes (*n_genes_by_counts*), total UMI counts (*total_counts*), and percentage of mitochondrial reads (*pct_counts_mt*) across all identified cell clusters after quality control filtering.

**B**, UMAP projections of all integrated cells from isografts (ISO_2M) and allografts (ALLO_1M and ALLO_2M).

**C**, UMAP plots showing normalized expression of selected canonical marker genes used for cell type identification.

**D**, Heatmap showing Pearson correlation coefficients between gene expression profiles of cell clusters.

**E**, UMAP plots showing expression of canonical CD4^+^ T cell marker genes.

**F**, Gating strategy for the flow cytometry analysis.

**G**-**H**, CD44 and CD62L expression defining Naive (CD44^−^CD62L^+^), Tcm (CD44^+^CD62L^+^), and Tem (CD44^+^CD62L^−^) subsets within CD4^+^ T cells across tissues from Allo-group mice.

Data are mean ± s.e.m. of biologically independent samples.

**
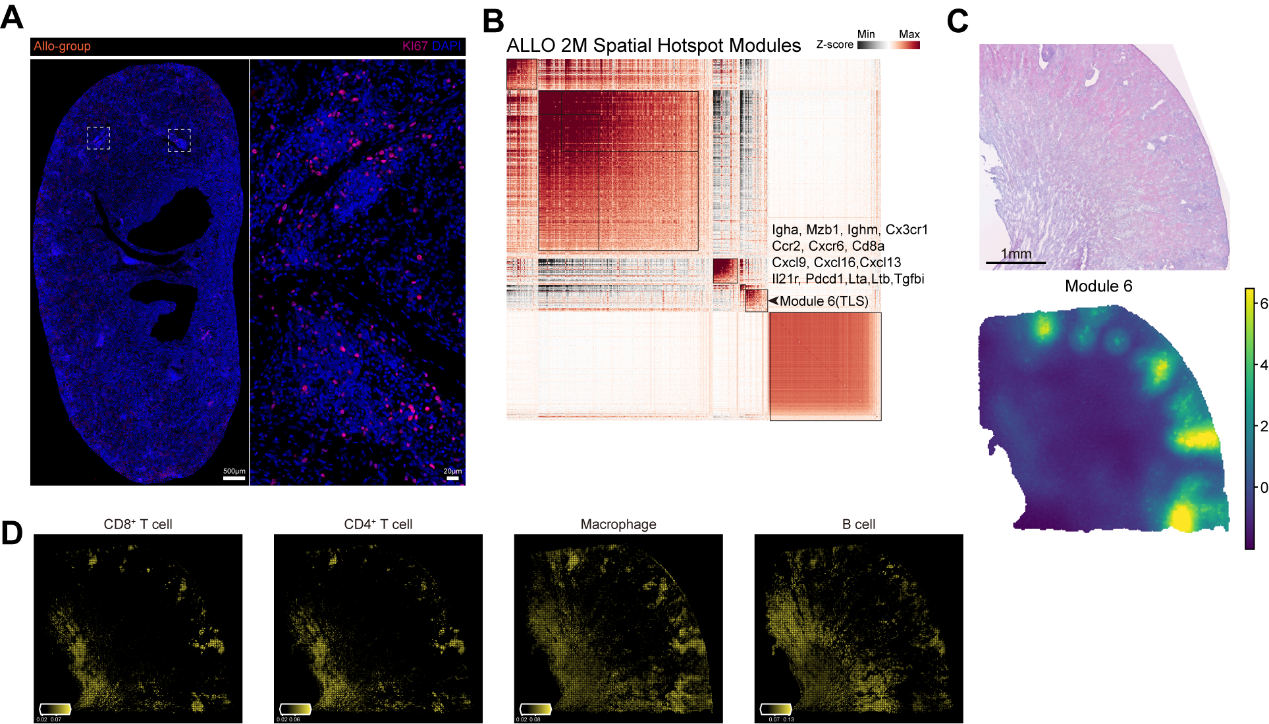
**

**Figure S3. Stereo-seq unveils TLS immune spots.**

**A**, Representative immunofluorescence images of KI67 staining in allograft sections. Scale bars, 500 μm and 20 μm.

**B**, Average expression levels for genes in the indicated spatial autocorrelated modules (section ALLO_2M).

**C**, H&E and spatial visualization of the module score (section ALLO_2M) for TLS area (module 6). Scale bars, 1 mm.

**D**, Spatial distribution of CD8^+^ T cell, CD4^+^ T cell, macrophages, and B cells across grafts inferred by Cell2location.


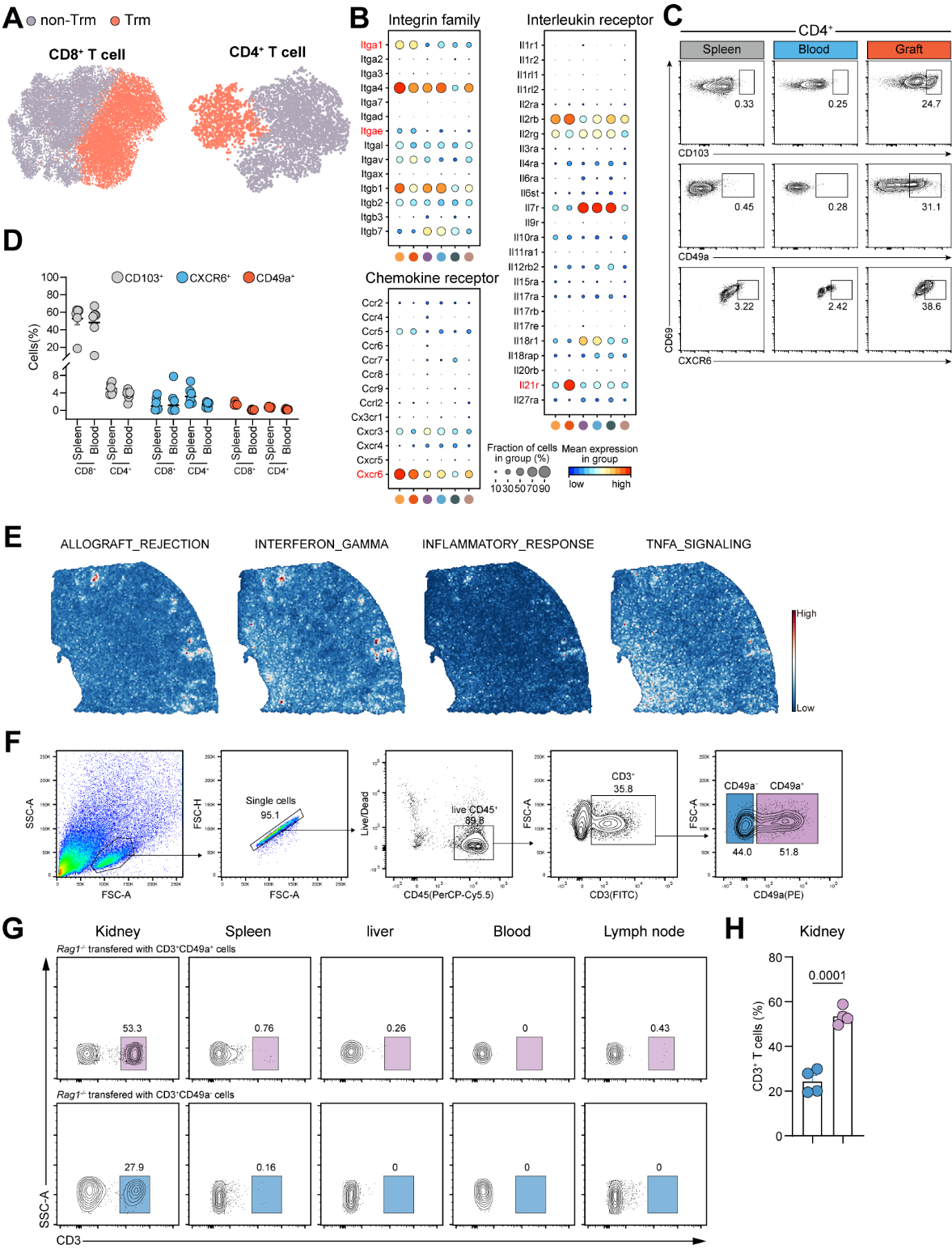


**Figure S4. Characterization and adoptive transfer of CD3^+^ T_RM_ cells.**

**A**, UMAP visualization of integrated CD8^+^ T cells (left) and CD4^+^ T cells (right), showing subclustering into T_RM_ (orange) and non-T_RM_ (grey) populations.

**B**, Dot plot showing the fraction of cells expressing (dot size) and mean expression level (color intensity) of selected integrin family, interleukin receptor, and chemokine receptor genes.

**C**, Representative flow cytometry plots showing the expression of CD103, CD49a, and CD69 versus CXCR6 on CD4^+^ T cells isolated from the spleen, blood, and kidney graft of allogeneic transplant recipient mice at day 30.

**D**, Frequencies of CD103^+^, CXCR6^+^, and CD49a^+^ cells among CD8^+^ and CD4^+^ T cells in the spleen and blood of wild-type mice.

**E**, Spatial activity patterns of selected signaling pathways on graft tissue section ALLO_2M, as inferred by PROGENy.

**F**, Representative flow cytometry plots showing the gating strategy used to define CD49a^+^or CD49a^−^ T cells for cell sorting.

**G**, Representative flow cytometry plots showing the frequency of transferred CD3^+^ T cells in the kidney, spleen, liver, blood, and lymph nodes of *Rag1*^-/-^ recipient mice at 30 days post-transfer (left). Quantification of the percentage of CD3^+^ T cells of transferred CD3^+^ T cells in the kidney (right).

Data are mean ± s.e.m. of biologically independent samples. P values are from a two-tailed unpaired Student’s t-test (H).


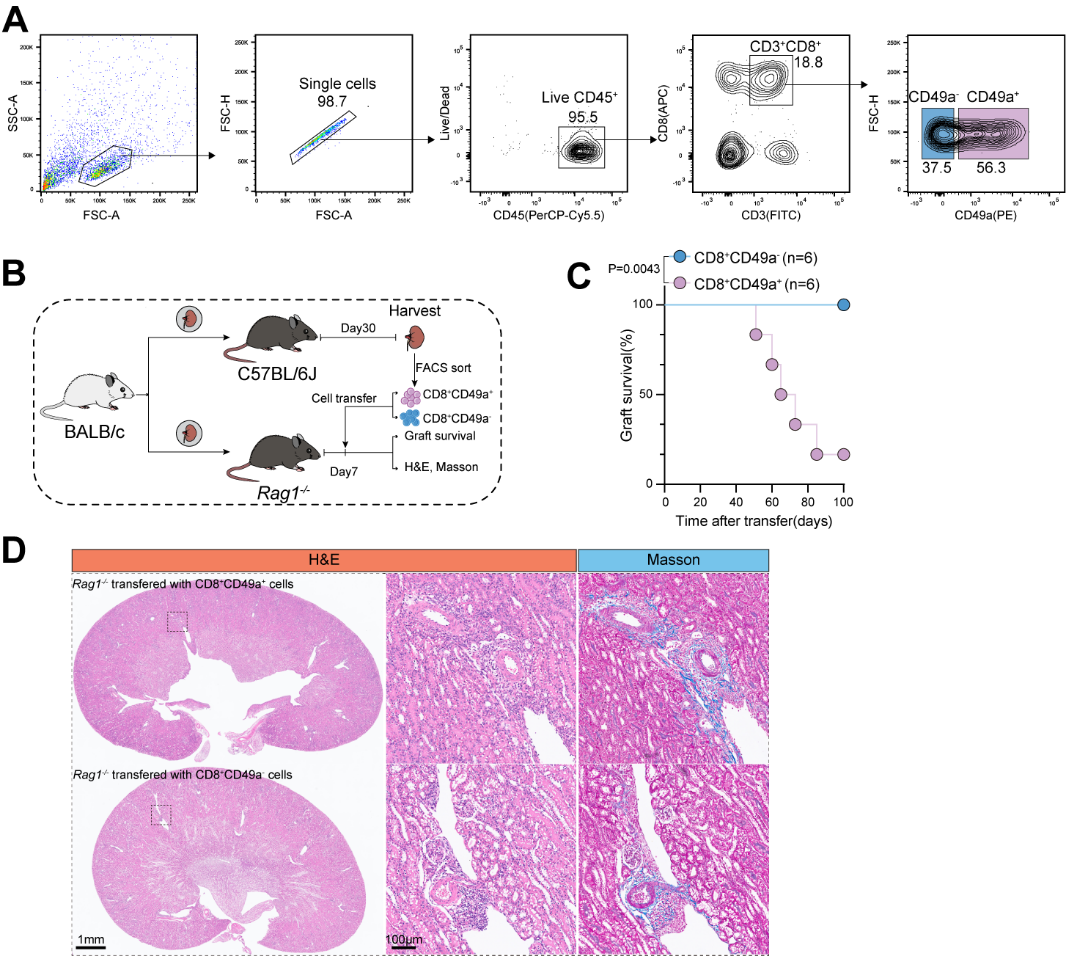


**Figure S5. Adoptive transfer and Characterization of CD8^+^ T_RM_ cells.**

**A**, Representative flow cytometry plots showing the gating strategy used to define CD49a^+^ CD8^+^or CD49a^−^CD8^+^ T cells for cell sorting.

**B**, Experimental design for adoptive transfer.

**C**, Kidney allograft survival following adoptive transfer of 1 × 10⁵ CD49a^+^ CD8^+^ or CD49a^−^CD8^+^ T cells. n = 6 mice per group.

**D**, Representative H&E and Masson's trichrome staining at day 40 post-transplantation. Scale bars, 1 mm and 100 μm.

Data are mean ± s.e.m. of biologically independent samples. P values are from log-rank test (C).


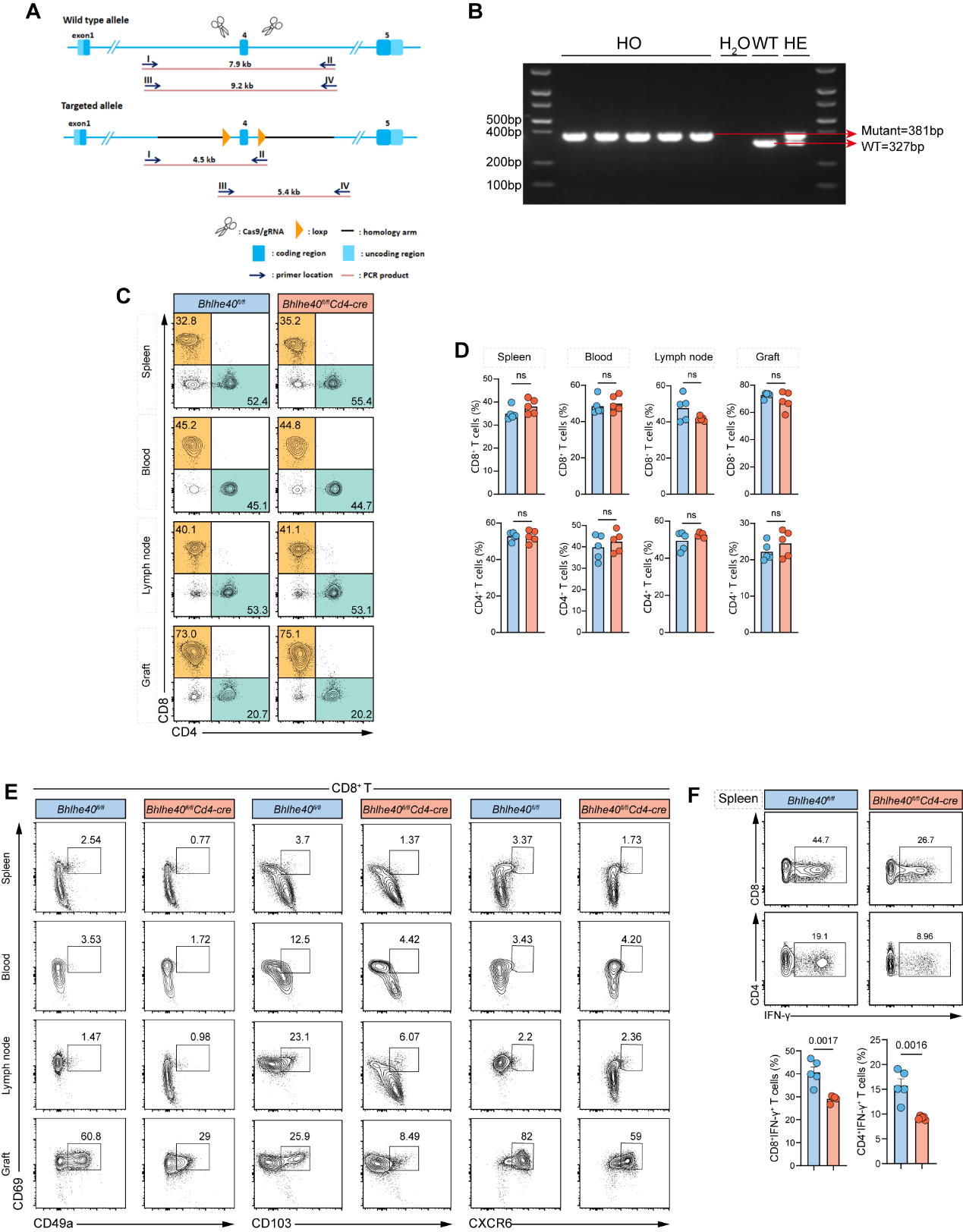


**Figure S6. Bhlhe40-deficiency** **mouse construction and analysis.**

**A**-**B**, The construction strategy of *Bhlhe40^fl/fl^* mice and representative genotyping images.

**C**, Flow plots and percentages of CD4^+^ and CD8^+^ T cells in *Bhlhe40^fl/fl^* and *Bhlhe40^fl/fl^ Cd4-Cre* (n = 5) mice across spleens, blood, lymph nodes, and grafts at day 30 post-transplantation.

**E**, Flow plots of CD69^+^CD49a^+^CD8^+^, CD69^+^CD103^+^CD8^+^, CD69^+^CXCR6^+^CD8^+^ in spleens, blood, lymph nodes, and grafts.

**F**, Flow plots and frequencies of IFN-γ^+^CD8^+^and IFN-γ^+^CD4^+^T cells in spleens (n = 5).

Data are mean ± s.e.m. of biologically independent samples. P values are from a two-tailed unpaired Student’s t-test (D, F).


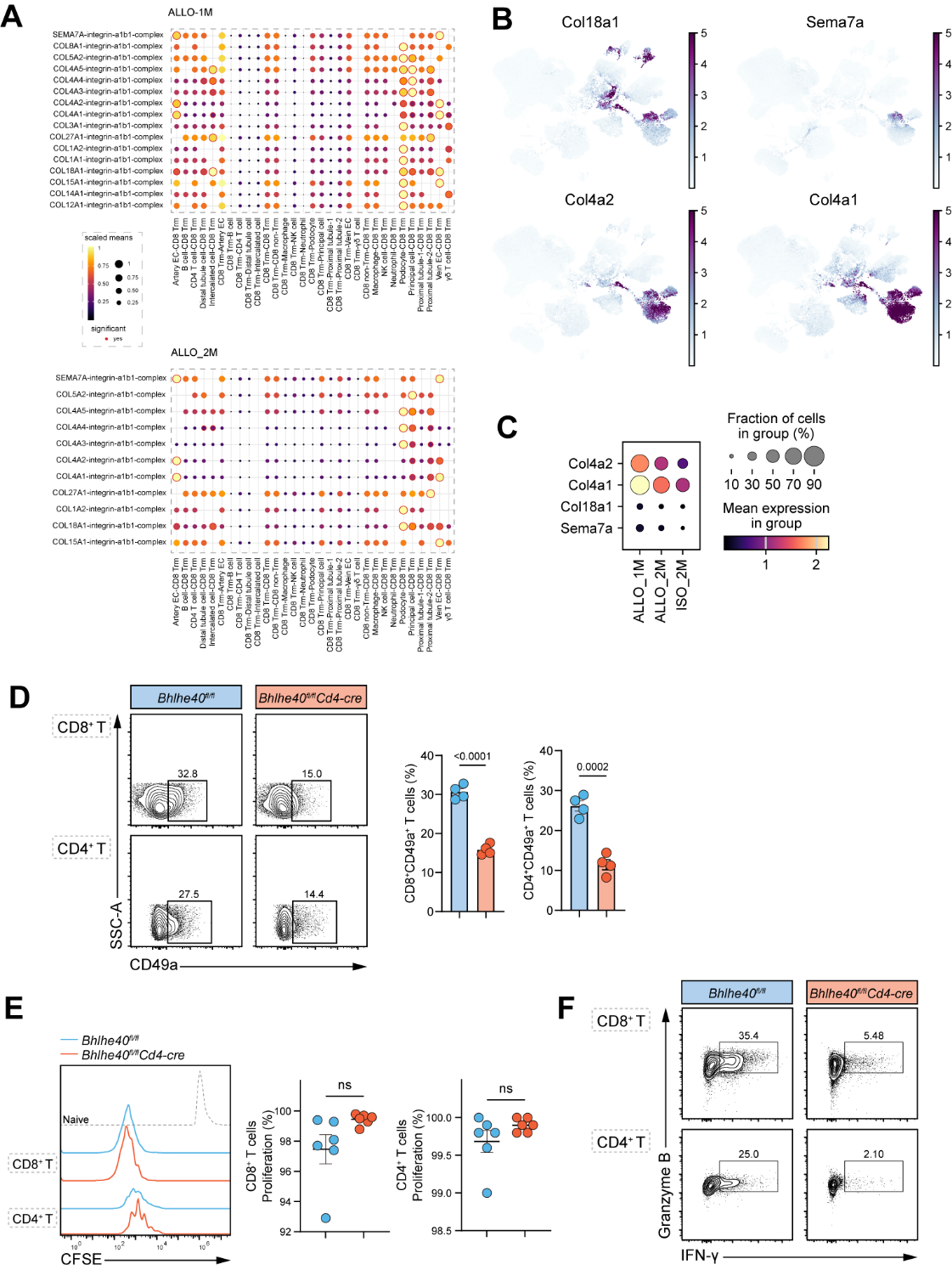


**Figure S7. T cells require TGF-β to establish tissue residency.**

**A**, Dot plots showing representative ligand–receptor pairs, including ligands interacting with the Itga1 receptor complex, at Allo_1M and Allo_2M group.

**B**, UMAP visualization of *Col18a1*, *Sema7a*, *Col4a2*, and *Col4a1* expression across all cells.

**C**, Dot plot illustrating the expression level (color) and proportion of expressing cells (dot size) for genes shown in (**B**) among EC cells (Artery and Vein EC) derived from different samples.

**D-F**, Flow plots and frequencies of CD49a^+^CD8^+^and CD49a^+^CD4^+^T cells following a 7-day in vitro culture with TGF-β (n = 4) (D). Flow cytometry histograms displaying the CFSE dilution of *Bhlhe40^fl/fl^* and *Bhlhe40^fl/fl^ Cd4-Cre* T cells (left); bar charts showing the percentages of proliferating CFSE^low^ cells (right) (E). Flow plots of GZMB^+^IFN-γ^+^CD8^+^ and GZMB^+^IFN-γ^+^CD4^+^T cells between *Bhlhe40^fl/fl^* and *Bhlhe40^fl/fl^ Cd4-Cre* (F).

Data are mean ± s.e.m. of biologically independent samples. P values are from a two-tailed unpaired Student’s t-test (D and E).


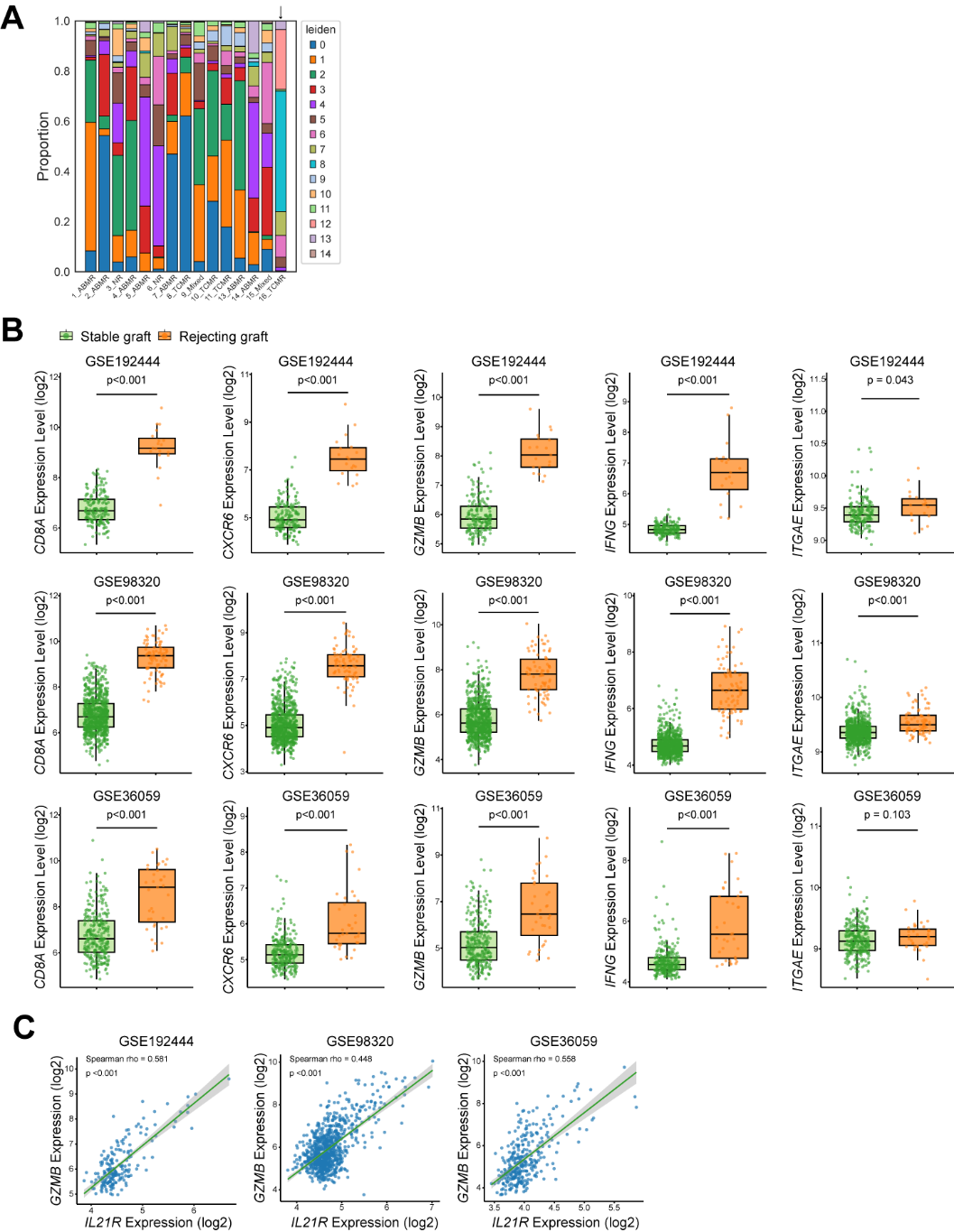


**Figure S8. Expression and correlation of T_RM_-associated and effector genes in human kidney allograft bulk RNA-seq datasets.**

**A**, Compositional analysis showing the proportion of each cell cluster within each individual biopsy sample.

**B**, Box plots showing log2 expression levels of *CD8A*, *CXCR6*, *GZMB*, *IFNG*, and *ITGAE* in stable grafts (green) versus rejecting grafts (orange) from three independent human kidney transplant cohorts: GSE19244 (top row), GSE98320 (middle row), and GSE36059 (bottom row). Statistical significance between stable and rejecting grafts for each gene was determined using a two-sided Wilcoxon rank-sum test. P-values are indicated on the plots.

**C**, Scatter plots showing the correlation between *IL21R* and *GZMB* log2 expression levels in the GSE19244 (left), GSE98320 (middle), and GSE36059 (right) cohorts. Spearman correlation coefficient (rho) and p-values are indicated. The green line represents the linear regression fit with 95% confidence interval (grey shaded area).
